# Heterogeneity in Slow Synaptic Transmission Diversifies Purkinje Cell Timing

**DOI:** 10.1523/JNEUROSCI.0455-24.2024

**Published:** 2024-08-15

**Authors:** Riya Elizabeth Thomas, Franziska Mudlaff, Kyra Schweers, William Todd Farmer, Aparna Suvrathan

**Affiliations:** ^1^Centre for Research in Neuroscience, Brain Repair and Integrative Neuroscience Program, Research Institute of the McGill University Health Centre, Montreal General Hospital, Montréal, Québec H3G 1A4, Canada; ^2^Departments of Neurology and Neurosurgery, McGill University, Montréal, Québec H3G 1A4, Canada; ^3^Pediatrics, McGill University, Montréal, Québec H3G 1A4, Canada; ^4^Integrated Program in Neuroscience, McGill University, Montréal, Québec H3A 1A1, Canada; ^5^Department of Pharmacology, University of North Carolina at Chapel Hill, Chapel Hill, North Carolina 27599; ^6^Neuroscience Center, University of North Carolina at Chapel Hill, Chapel Hill, North Carolina 27599

**Keywords:** cerebellum, metabotropic glutamate receptor, Purkinje cell, synapse

## Abstract

The cerebellum plays an important role in diverse brain functions, ranging from motor learning to cognition. Recent studies have suggested that molecular and cellular heterogeneity within cerebellar lobules contributes to functional differences across the cerebellum. However, the specific relationship between molecular and cellular heterogeneity and diverse functional outputs of different regions of the cerebellum remains unclear. Here, we describe a previously unappreciated form of synaptic heterogeneity at parallel fiber synapses to Purkinje cells in the mouse cerebellum (both sexes). In contrast to uniform fast synaptic transmission, we found that the properties of slow synaptic transmission varied by up to threefold across different lobules of the mouse cerebellum, resulting in surprising heterogeneity. Depending on the location of a Purkinje cell, the time of peak of slow synaptic currents varied by hundreds of milliseconds. The duration and decay time of these currents also spanned hundreds of milliseconds, based on lobule. We found that, as a consequence of the heterogeneous synaptic dynamics, the same brief input stimulus was transformed into prolonged firing patterns over a range of timescales that depended on Purkinje cell location.

## Significance Statement

The cerebellum is separated into functionally distinguished lobules, yet the lobules have a repeated and similar pattern of connectivity. Thus, it remains unclear how cells and circuits manage to perform the diverse functions that the cerebellum supports. Our results demonstrate that cerebellar Purkinje cells have synapses with strikingly different properties across lobules. This synaptic diversity drives heterogeneously timed output responses to the same input. Our results lay the framework for elucidating heterogeneity in the intracellular signaling pathways of these synapses. Overall, we demonstrate a heterogeneity of synaptic timing properties that can serve to diversify information processing across functionally different regions of the cerebellum.

## Introduction

The cerebellum supports a number of functions, far beyond the motor domain (Stoodley et al., 2012; Witter and De Zeeuw, 2015a). Yet, cerebellar cortical microcircuits are known for their uniformity, consisting of repeated stereotyped modules (Apps and Hawkes, 2009; Ruigrok, 2011; Cerminara et al., 2015; Valera et al., 2016; Apps et al., 2018). The homogeneity of cerebellar architecture has led to the assumption that the computations performed are similar across regions (Schmahmann, 2010), despite their diverse functional roles. However, more recent discoveries demonstrated a marked degree of molecular and cellular heterogeneity across cerebellar lobules (Zhou et al., 2014, 2015; Cerminara et al., 2015; Tsutsumi et al., 2015; Witter and De Zeeuw, 2015b; Suvrathan et al., 2016; Nguyen-Minh et al., 2019). The functional impact of this heterogeneity is not well understood.

The properties of Purkinje cells, the output neurons of the cerebellar cortex, vary across regions, e.g., with respect to dendritic integration (Eccles et al., 1967), firing patterns (Zhou et al., 2014, 2015), and axonal output (Voogd, 2011). However, the potential heterogeneity of Purkinje cell inputs is not well explored.

The inputs to Purkinje cells are well known to carry diverse information (Brodal and Bjaalie, 1997). For instance, parallel fibers (PFs) can encode sensory (Bosman et al., 2010; Shimuta et al., 2020), motor (Wiestler et al., 2011; Proville et al., 2014), or reward (Wagner et al., 2017) features. Thus, there is reason to hypothesize that there is heterogeneity in these inputs.

The cerebellar cortex is patterned into parasagittal microzones of Purkinje cells that are defined by their connectivity and correlate with their molecular identity (Cerminara et al., 2015). The glycolytic enzyme aldolase C or zebrin II is a well-investigated marker of Purkinje cell heterogeneity, which patterns the cerebellar cortex into parasagittal zebra-like bands (Apps et al., 2018). Zebrin bands receive similar input information and send similar output information (Sugihara and Shinoda, 2004; Voogd and Ruigrok, 2004; Pijpers et al., 2006; Apps and Hawkes, 2009; Ruigrok, 2011), although functional units within the cerebellum can also cross zebrin boundaries (Graham and Wylie, 2012). Zebrin identity correlates with some forms of molecular heterogeneity, including components of the metabotropic glutamate receptor 1 (mGluR1) signaling cascade (Mateos et al., 2001; Furutama et al., 2010; Wu et al., 2019). These molecular differences in Purkinje cells with different zebrin identities have been previously shown to correlate with differences in firing rate (Zhou et al., 2014) and with plasticity at PF synapses (Wadiche and Jahr, 2005).

At PF to Purkinje cell synapses, excitatory synaptic transmission is composed of a fast, α-amino-3-hydroxy-5-methyl-4-isoxazolepropionic acid receptor (AMPAR)–dependent input, as well as a slower mGluR1-dependent component (Batchelor et al., 1994; Batchelor and Garthwaite, 1997; Tempia et al., 1998). The metabotropic arm of PF input triggers an intracellular signaling cascade and a slow excitatory postsynaptic current (slow EPSC or sEPSC) through the canonical transient receptor potential channel TRPC3 (Hartmann et al., 2008, 2011; Hartmann and Konnerth, 2015). mGluR1 signaling (Aiba et al., 1994; Ichise et al., 2000; Lüscher and Huber, 2010) and TRPC3-dependent currents (Hartmann et al., 2008, 2011; Becker, 2014; Cole and Becker, 2023) are both essential for normal cerebellar function. However, the heterogeneity of mGluR1-mediated slow synaptic transmission across lobules has never yet been investigated.

Here, we tested the hypothesis that slow synaptic transmission at PF synapses varies across different lobules of the cerebellum. To do so, we measured synaptic currents at PF inputs to Purkinje cells in acute mouse brain slices. We found that the properties of slow synaptic currents varied up to threefold across different cerebellar lobules. As a consequence, PF inputs were transformed into a diverse range of prolonged firing outputs that depended on Purkinje cell location. This previously unappreciated heterogeneity in slow synaptic transmission thus diversifies Purkinje cell firing dynamics.

## Materials and Methods

All experiments were done in accordance with the policies of the Canadian Council on Animal Care, using protocols approved by the Montreal General Hospital Facility Animal Care Committee, using C57BL/6J mice of both sexes from the Jackson Laboratory (Strain number 000664). 

### Ex vivo slice electrophysiology

Mice (21–40 d) of both sexes were used for all slice electrophysiology experiments. Mice were maintained on an inverted day–night cycle, with *ad libitum* access to food and water. The cerebellum was dissected, and 300-µm-thick acute cerebellar slices were prepared in the sagittal (for vermis recordings) or coronal orientation (for flocculus recordings) using a Leica VT1200S vibratome in ice-cold aCSF [containing the following (in mM): 119 NaCl, 2.5 KCl, 1 NaH_2_PO_4_, 26.2 NaHCO_3_, 1.3 MgCl_2_, 2.5 CaCl_2_, 10 d-glucose, equilibrated with carbogen (95% O_2_, 5% CO_2_, Linde Canada)] or in ice-cold sucrose cutting solution [containing the following (in mM): 200 sucrose, 2.5 KCl, 1 NaH_2_PO_4_, 26.2 NaHCO_3_, 1.3 MgCl_2_, 2.5 CaCl_2_, 20 d-glucose]. The slices were allowed to recover at ∼35°C for 15–25 min in aCSF with constant carbogen bubbling and then at room temperature for 1 h.

**Figure 1.**
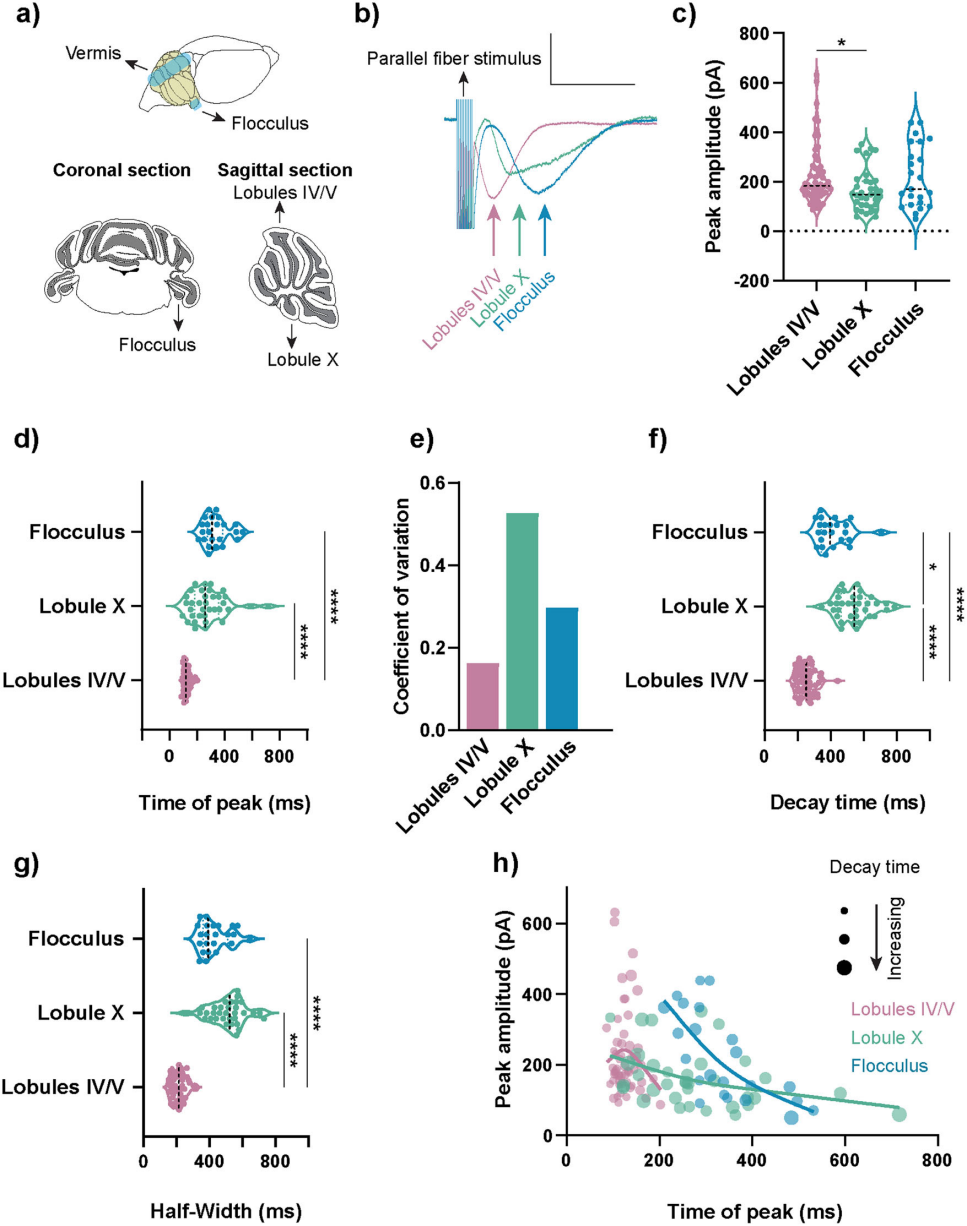
The slow EPSC (sEPSC) was heterogenous across cerebellar lobules. ***a***, Schematic representation of a mouse brain highlighting the recorded regions: the cerebellar flocculus and vermis. Parasagittal acute cerebellum slices were used for recordings in vermis lobules IV/V and X, while coronal sections were used for flocculus recordings. ***b***, Representative sEPSC traces depict a delayed time of peak in lobule X and the flocculus compared with lobules IV/V. Scale bar, 100 pA, 500 ms. ***c***, Peak amplitudes of sEPSCs were similar across lobules. *p < 0.05, Kruskal–Wallis analysis of variance on ranks followed by Dunn's multiple-comparisons test. ***d***, sEPSC time of peak was earlier in lobules IV/V in comparison with lobule X and the flocculus. Kruskal–Wallis analysis of variance on ranks followed by Dunn's multiple-comparisons test (****p < 0.0001). ***e***, High heterogeneity in the time of peak within lobule X and the flocculus compared with lobules IV/V is demonstrated by higher coefficients of variation. ***f***, Lobule X sEPSCs exhibited the longest 90–10% decay time. Both lobule X and flocculus sEPSCs had longer decay times than those from lobules IV/V. *p < 0.05, ****p < 0.0001, Kruskal–Wallis analysis of variance on ranks followed by Dunn's multiple-comparisons test. ***g***, A longer half-width sEPSC was observed in lobule X and the flocculus compared with lobules IV/V (****p < 0.0001, Kruskal–Wallis analysis of variance on ranks followed by Dunn's multiple-comparisons test). ***h***, sEPSCs with similar peak amplitudes in lobules IV/V, lobule X, and the flocculus had different times of peak. Bubble size represents the decay time of the sEPSC. Data from each lobule were fit in an assumption-free manner with a spline in equivalent color. Violin plots show median and quartiles. (**c**–**f**, ***h***: lobules IV/V = 56 cells from 41 mice, lobule X = 35 cells from 27 mice, flocculus = 23 cells from 14 mice. **g**: lobules IV/V = 42 cells from 33 mice, lobule X = 33 cells from 26 mice, flocculus = 23 cells from 14 mice). Please refer to Extended Data Figures 1-1–1-3 for further information. Additional details on statistics are presented in Materials and Methods and Extended Data Table 1.

Purkinje cells were visualized under an Olympus BX61WI upright microscope using differential interference contrast optics. Patch electrodes (3–6 MΩ) were pulled from borosilicate glass and filled with an internal solution containing either one of the two recipes for both voltage and current-clamp experiments [containing the following (in mM): Recipe 1, 135 potassium gluconate, 7 NaCl, 2 MgATP, 0.3 NaGTP, 10 HEPES, 0.5 EGTA,10 phosphocreatine di(tris) salt (pH 7.2); Recipe 2, 128 potassium gluconate, 4 KCl, 10 HEPES, 10 sodium creatine phosphate, 4 MgATP, 0.3 NaGTP (pH 7.3)]. Recordings made with the two internal solutions were identical and have been merged. For the experiments in Extended Data Figure 6-1, the internal solution contained 10 mM EGTA and 1 mM BAPTA with modifications to Recipe 1 containing (in mM) 130 potassium gluconate, 1 BAPTA, 7 NaCl, 2 MgATP, 0.3 NaGTP, 10 HEPES, 10 EGTA, and 10 phosphocreatine di(tris) salt (pH 7.2). For cell-attached recordings, the recording electrode was filled with NaCl (162.5 mM) solution (Perkins, 2006). Stimulation of the parallel fibers was performed using bipolar stimulating electrodes made from theta glass that were positioned in the outer molecular layer to trigger a slow EPSC or increase in firing. aCSF contained 50 µM picrotoxin and 5 µM NBQX for all recordings (except for Extended Data Fig. 1-1, where only picrotoxin was added, and Fig. 10, where no blockers were added). All whole-cell patch-clamp recordings were performed at 29–30°C. For cell-attached recordings, the temperature was maintained at 33 ± 1°C. Signals were acquired using a MultiClamp 700B amplifier at 10–100 kHz for the slow EPSC and 50 kHz for spiking experiments. The slow EPSC traces were averaged and filtered using a Bessel (eight-pole) filter with a low-pass filter of 1 kHz. Only recordings with series resistance <25 MΩ were included. Both input and series resistance were monitored for stability and discarded if they changed >20%. Slow EPSCs were elicited using 10 PF stimulation at 100 Hz for all experiments except Figures 3, 4, 9 and 10, where the frequency and number of PF stimulation were varied, as described in the figure legends. For voltage-clamp recordings, the cells were held at −70 mV. For the whole-cell spiking experiments (Fig. 9*a–i*), the slow EPSC traces were first recorded in voltage-clamp configuration and consequently switched to current-clamp with sufficient current injection to keep the cells below the threshold for spiking (between –49 and −57 mV at baseline. In only one flocculus cell was the cell spiking during the pre-stimulus baseline). For Figures 3 and 4, some cells that contributed to the data came from the same cells recorded in Figures 1, 9*a–i*, or 5. Cell-attached recordings were analyzed by normalizing the instantaneous firing frequency according to the following equation: (Inst. Freq. − Mean baseline Inst. Freq.) / (Inst. Freq. + Mean baseline Inst. Freq.).

10.1523/JNEUROSCI.0455-24.2024.f1-1 Figure 1-1 **AMPAR-mediated synaptic responses in lobules IV/V and flocculus were indistinguishable.** a) Representative traces of AMPAR-mediated EPSC from lobules IV/V and the flocculus. Scale bar 100 pA, 50 ms. Peak amplitude (b) and time of peak (c) were the same in lobules IV/V and the flocculus, in contrast to the time of peak of sEPSCs. Violin plots show median and quartiles. (Fig 1-1 b,c: Lobules IV/V = 8 cells from 4 mice, flocculus = 5 cells from 5 mice.) Download Figure 1-1, TIF file.

**Figure 2.**
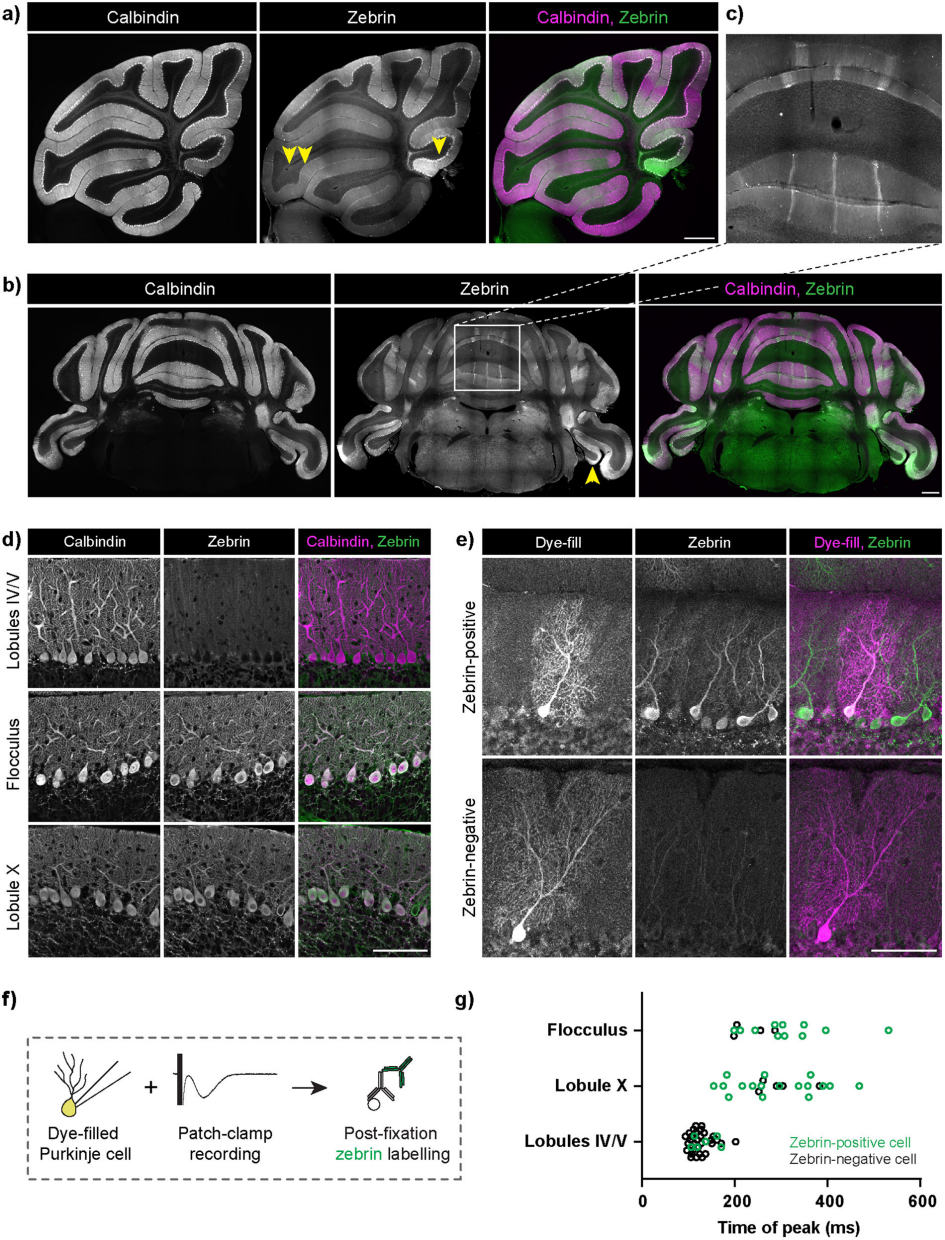
Zebrin/aldolase C expression did not predict sEPSC peak latency. ***a***, Stitched sagittal sections of the mouse vermis, immunolabelled with anti-calbindin (which marks all Purkinje cells) and anti-zebrin antibodies. Rightmost panel, merged. Single arrowhead marks lobule X, double arrowhead marks lobules IV/V. Scale bar, 500 µm. ***b***, Stitched coronal sections of the mouse cerebellum, immunolabelled with anti-calbindin and anti-zebrin antibodies. Rightmost panel, Merged. The single arrowhead marks the flocculus. Scale bar, 500 µm. ***c***, Inset illustrates zebrin stripes. ***d***, Zebrin-negative Purkinje cells in lobules IV/V and zebrin-positive Purkinje cells in lobule X and the flocculus. Scale bar, 100 µm. Images are maximum intensity projections of confocal stacks. ***e***, Representative image of a 300-µm-thick cerebellar section with the Alexa Fluor 488 dye-filled Purkinje cell, with the section retrospectively stained with anti-zebrin antibody. The top row demonstrates a dye-filled cell that is zebrin-positive, and the bottom row demonstrates one that is zebrin-negative. Images are maximum intensity projections of confocal stacks. Scale bar, 100 µm. ***f***, Schematic representing dye-fill of the whole-cell patch-clamped Purkinje cell from which sEPSCs were measured, followed by immunolabelling with anti-zebrin antibody. ***g***, Zebrin identity did not predict the time of peak of sEPSC, across regions. Most dye-filled lobules IV/V cells were zebrin-negative (black hollow circles), and most dye-filled lobule X and flocculus cells were zebrin-positive (green hollow circles). However, even zebrin-positive cells (green hollow circles) in lobules IV/V had a characteristically shorter time of peak, and zebrin-negative cells (black hollow circles) in lobule X and the flocculus had a delayed time of peak. (**g**: lobules IV/V = 37 cells from 28 mice, lobule X = 22 cells from 18 mice, flocculus = 15 cells from 14 mice).

10.1523/JNEUROSCI.0455-24.2024.f1-2 Figure 1-2 **Lobule-specific synaptic dynamics were not sensitive to Purkinje cell dialysis.** a) Time of peak of a subset of sEPSCs from Figure 1, across lobules IV/V, lobule X, and the flocculus, recorded within 10-20 min after break-in to whole-cell configuration, demonstrated shorter time of peak in lobules IV/V. b) Peak amplitude of sEPSCs was similar across regions, even for the subset of cells. c) Decay time of sEPSCs was shorter in lobules IV/V, in comparison to lobule X and the flocculus, even for the subset of cells. d) Half-widths of sEPSCs were shorter in lobules IV/V, in comparison to lobule X and the flocculus, even for the subset of cells. Statistical comparisons: (a,b,c,d)*p < 0.05, ***p < 0.001, ****p < 0.0001, Kruskal-Wallis test followed by Dunn’s multiple comparisons test. Violin plots show median and quartiles. (Fig 1-2 a-c: lobules IV/V = 31 cells from 27 mice, lobule X = 27 cells from 22 mice; flocculus = 14 cells from 11 mice. Fig 1-2 d: lobules IV/V = 23 cells from 21 mice, lobule X = 25 cells from 21 mice; flocculus = 14 cells from 11 mice.) Download Figure 1-2, TIF file.

**Figure 3.**
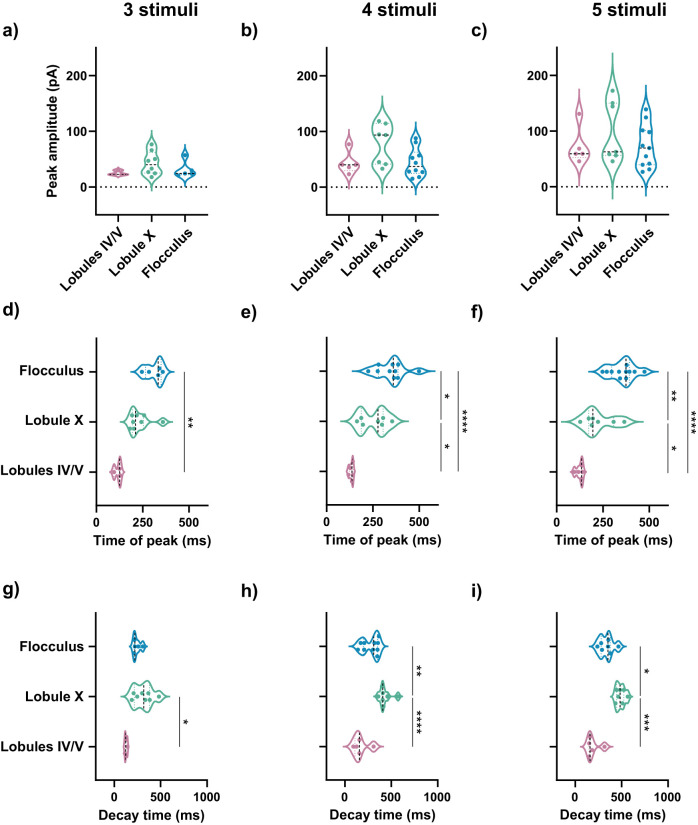
sEPSC heterogeneity did not depend on the number of PF stimulations. ***a–c***, Peak amplitude of sEPCS were similar in lobules IV/V, lobule X, and the flocculus in response to three, four, and five PF stimulations, respectively at 100 Hz. ***d–f***, sEPSC time of peak was faster in lobules IV/V in response to three, four, or five PF stimulations at 100 Hz. ***g–i***, sEPSC decay time was longer in lobule X than lobules IV/V in response to three PF stimulations at 100 Hz and longer in lobule X in comparison with both lobules IV/V and the flocculus in response to four or five PF stimulations at 100 Hz. Statistical comparisons: (***b***, ***e***, ***f***, ***h***) *p < 0.05, **p < 0.01, ****p < 0.0001, ordinary one-way ANOVA followed by Tukey's multiple-comparison test. ***a***, ***c***, ***d***, ***g***, ***i***, *p < 0.05, **p < 0.01, ***p < 0.001, Kruskal–Wallis test followed by Dunn's multiple-comparisons test. Violin plots show median and quartiles. (***a***, ***d***, ***g***: lobules IV/V = 3 cells from 3 mice, lobule X = 8 cells from 3 mice, flocculus = 5 cells from 2 mice. ***b***, ***e***, ***h***: lobules IV/V = 5 cells from 3 mice, lobule X = 7 cells from 3 mice, flocculus = 10 cells from 5 mice. ***c***, ***f***, ***i***: lobules IV/V = 5 cells from 3 mice, lobule X = 7 cells from 3 mice; flocculus = 11 cells from 5 mice). Additional details on statistics are in Extended Data Table 1.

10.1523/JNEUROSCI.0455-24.2024.f1-3 Figure 1-3 **Animal sex did not determine lobule-specific synaptic dynamics.** a) Subset analysis of data in Figure 1 demonstrated that the sEPSC time of peak did not differ across sexes, nor did peak amplitude (b), decay time (c), or half-width (d). Statistical comparisons: (a,b,c,d) *p < 0.05, ***p < 0.001, ****p < 0.0001, Kruskal-Wallis test followed by Dunn’s multiple comparisons test. (Fig 1-3 a-c: Lobules IV/V male = 23 cells from 17 mice, Lobules IV/V female = 27 cells from 20 mice, lobule X male = 18 cells from 13 mice; lobule X female = 16 cells from 13 mice, flocculus male = 16 cells from 8 mice, flocculus female = 5 cells from 4 mice; Fig 1-3 d: Lobules IV/V male = 18 cells from 13 mice, Lobules IV/V female = 19 cells from 16 mice, lobule X male = 16 cells from 12 mice; lobule X female = 16 cells from 13 mice, flocculus male = 16 cells from 8 mice, flocculus female = 5 cells from 4 mice.) Download Figure 1-3, TIF file.

### Fixed tissue preparation and immunofluorescence

Sedated mice underwent intracardial perfusion with ice-cold PBS followed by 4% PFA in PBS. Brains were postfixed in a 4% PFA solution at 4°C overnight. Subsequently, agarose-embedded cerebella were cut into 50 μm slices, in either a sagittal or coronal orientation, using a Vibratome 1000 Plus. For immunofluorescent labeling, slices were incubated with primary antibodies for 2 d at 4°C followed by secondary antibody incubation for 90–120 min at room temperature. Blocking of endogenous immunoglobulins with Fab fragment antibodies was performed when using mouse primary antibody. For immunostaining of 300-µm-thick slices, post-electrophysiology, and dye-fill (Fig. 2*e–g*), the slices were fixed in PFA for 1 to 4 h. The immunostaining protocol was the same as for 50 µm slice staining except for a 3 d incubation time with primary antibody (Table 1).

**Table 1.** Antibodies

	Dilution (for 50 µm slices)	Dilution (for 300 µm slices)	Vendor and catalog number	Figures
Primary antibodies				
1) Mouse anti-aldolase C antibody (4A9)	1:300	1:100	Abcam (ab190368)	2
2) Rabbit anti-calbindin D28K	1:500	N/A	Thermo Fisher Scientific (PI711443)	2
3) Chicken anti-calbindin D28K	1:2,000	N/A	Thermo Fisher Scientific (PA5143561)	2, 8
4) Rabbit anti-TRPC3	1:200	N/A	Cell Signaling Technology (77934S)	8
5) Mouse anti-mGluR1α	1:250	N/A	BD Biosciences (556389)	8
Secondary antibodies				
1) Goat anti-mouse Alexa Fluor 568	1:1,000	1:1,000	Thermo Fisher Scientific (A-1004)	2
2) Goat anti-rabbit Alexa Fluor 488	1:1,000	N/A	Thermo Fisher Scientific (A-1008)	2
3) Goat anti-mouse Alexa Fluor 488	1:500	N/A	Thermo Fisher Scientific (A-1029)	2, 8
4) Goat anti-rabbit Alexa Fluor 647	1:500	N/A	Thermo Fisher Scientific (A-32733)	8
5) Goat anti-chicken Alexa Fluor 647	1:1,000	N/A	Thermo Fisher Scientific (A-1449)	2
6) Goat anti-chicken Alexa Fluor 405	1:1,000	N/A	Thermo Fisher Scientific (A-8260)	8

The Fab fragment used was AffiniPure Fab Fragment Donkey Anti-Mouse by Jackson Immunoresearch (715-007-003) at 1:50, 1:150, or 1:200 dilution.

For Figures 2*d* and 8, prior to staining, slices were subjected to an antigen retrieval procedure, in which the slices were boiled at 90°C for 10 min in 10 mM trisodium citrate buffer (pH 8.5), followed by a 20 min cooldown period. For Figures 2, *a*, *b*, *c*, *e*, and *g*, and 8, blocking was performed in 1× PBS, pH 7.4, 0.4% Triton X-100, 5% BSA, and 0.05% sodium azide. For Figure 2*d*, 5% BSA was substituted with 8% heat-inactivated NGS.

Primary and secondary antibodies (Table 1) were diluted in the respective blocker solutions.

Epifluorescence images were captured on two different microscopes. Either we used a fully automated epifluorescence microscope system (Ti2-E, Nikon), equipped with a multispectral LED light source (SpectraX, Lumencor) and 10× Plan Apo 0.45 NA objective, a motorized emission filter wheel (HS-1025, FLI), a motorized stage, and an sCMOS camera (ORCA Fusion BT, Hamamatsu) controlled by Nikon Elements AR. Images were acquired as single images or as multipoint images that were stitched together using Nikon Elements AR's “large image” feature. Alternatively, images were acquired on an Olympus FV1000 laser scanning microscope, with a 20× or 40× lens, and acquired using Fluoview software (Olympus). Post–patch-clamp recording zebrin labeling was quantified by taking a ratio of fluorescent intensity within the Purkinje cell to the background fluorescence in the neighboring granule cell layer using Fiji (ImageJ). Only cells with a ratio >2 were considered zebrin-positive. Those with a ratio <1.7 were considered zebrin-negative, and those in between were considered uncertain and were therefore not included in the analysis shown in Figure 2.

### Quantitative reverse transcription polymerase chain reaction (qRT-PCR)

Cerebella of 26–33-day-old mice of both sexes were extracted in ice-cold aCSF bubbled with 95% O_2_ and 5% CO_2_, with lobules of interest (lobules IV/V, lobule X, and flocculus) dissected under a dissection microscope and immediately snap frozen. Each biological sample was prepared by clubbing the lobule of interest from three mice. RNA extraction was performed using the RNeasy Mini Kit (Qiagen, 74104) following the manufacturer's instructions. RNA was then transcribed into cDNA using the QuantiTect Reverse Transcription Kit (Qiagen, 205313). qPCR was performed on a StepOnePlus Real-Time PCR System (Applied Biosystems by Thermo Fisher Scientific) using Fast SYBR Green Master Mix (Applied Biosystems, 4385612). Relative expression levels of each target transcript were determined by the ΔΔCт method (Livak and Schmittgen, 2001) using RPL13 mRNA levels as a reference. qPCR reactions were run as technical triplicates in 10 μl reactions (96-well format). The thermal cycling was performed with the following settings: holding stage, 20 s at 95°C; and cycling stage, 40 cycles of 3 s at 95°C and 30 s at 62°C. Melt curves were generated by heating from 62°C to 95°C at a temperature increment of 0.3°C.

### Primer sequences

A list of primer sequences is presented in Table 2.

**Table 2.** Primer sequences

Primer name	Forward (5′-3′)	Reverse (5′-3′)
RPL13	GGCCAGAGTTATCACAGAAGAA	TTGCTCGGATGCCAAAGA
Total TRPC3	GATCGAGGATGACAGTGATGTAG	GACTGAAGGGTGGAGGTAATG
TRPC3b	TCCAAATGCAGGAGGAGAAG	CGAGTTAGACTGTGTGAAGAGG
TRPC3c	GTAACTCAAAGTCCAGGCAGATA	TCAGTTCACCTTCATTCACCTC
Total mGluR1	TCTGTGATTCCCTGTGTTTAGG	CGTCTCGCTTCTCTCTTTACTC
mGluR1a	AGGGAACGCCAATTCTAACG	CCACACATGCTGTCCCTT
mGluR1b	CAAGAAGAGGCAGCCAGAA	GCCGTTAGAAACATTCACTGC

### Statistical analysis

Slice physiology data were analyzed in pCLAMP 11 (Clampfit), and the statistical tests were performed using GraphPad Prism, IBM SPSS, or the language R, except for Levene’s test, which was performed on the JASP statistics program.

The data were tested for normality using the Shapiro–Wilk test and for equality of variances using Levene's test. If the data were normally distributed and had equal variances, then it was analyzed using ordinary one-way ANOVA followed by Tukey's multiple-comparisons test. If either one of the assumptions was violated, the Kruskal–Wallis test followed by Dunn's multiple-comparisons test was used. For paired comparisons, paired *t* tests were used if the data passed the normality test using the Shapiro–Wilk test. If not, the Wilcoxon matched-pair signed rank test was used. For Extended Data Figure 1-1, an unpaired *t* test was used following the normality check using the Shapiro–Wilk test.

In several experiments, more than one cell came from a single mouse. Therefore, we fitted a linear mixed-effects (LME) model (both in SPSS and R) with the mouse as a random effect for the fixed effect of each variable we assessed (Yu et al., 2022). Even when accounting for more than one cell coming from a single mouse, analysis of data in Figure 1 demonstrates that the peak amplitude is significantly different across lobules, with a Bonferroni’s pairwise comparison indicating a significant difference between lobules IV/V and lobule X (*p* < 0.05). In addition, the time of peak of all three lobules was also significantly different from each other (lobules IV/V vs flocculus and lobule X = *p* < 0.001; lobule X vs flocculus = *p* < 0.05). The half-width was also significantly different across lobules (pairwise comparison: lobules IV/V vs lobule X and flocculus = *p* < 0.001; lobule X vs flocculus = *p* < 0.01). Thus, the heterogeneity in sEPSCs held true even when accounting for the effect of mouse identity. We tried to fit LME models to all datasets in the paper, but only in a subset of figures was the LME able to estimate a random effect coming from the mouse identity. Importantly, in no case did the LME contradict any of the significant effects we describe.

Details on statistical analysis are provided in each figure legend, with additional information in Extended Data Table 1.

10.1523/JNEUROSCI.0455-24.2024.t1-1 Table 1 Details on statistical analyses. Download Table 1, DOCX file.

### Single-nucleus RNA-seq analysis

The processed single-cell RNA-seq data from Kozareva et al., 2021 was filtered to contain only the 16,634 Purkinje cells using Seurat 5.0.1 in R 4.3.2. The slots containing metadata, raw counts, normalized, and scaled data were copied from the author's Seurat 2.3.4 object found here https://singlecell.broadinstitute.org/single_cell/study/SCP795/a-transcriptomic-atlas-of-the-mouse-cerebellum to create a new Seurat v5 compatible object. Variable features, principal components, and UMAP dimension reductions were then recalculated using the respective Seurat functions on the Purkinje-only data. Plots were produced using the DimPlot and DotPlot functions of Seurat and ggplot2. Differential gene expression analysis was performed using the FindMarkers function of Seurat. To determine if genes were differentially expressed between lobules, the Seurat function FindMarkers with default parameters (Wilcoxon rank sum test with Bonferroni’s correction for multiple tests) was used pairwise between the three lobes/regions of interest on all detected genes. Genes with an adjusted *p*-value < 0.05 were deemed to be significantly differentially expressed between tested lobes.

Reagents were obtained from the following vendors: NBQX (Sigma-Aldrich N-183 or Abcam ab120046), CPCCOEt (Sigma-Aldrich SML1124 or Abcam ab120060), Alexa Fluor 488 hydrazide (Thermo Fisher Scientific A10436). Picrotoxin (P1675), genistein (G6776), and SKF-96365 (S7809) were all obtained from Sigma-Aldrich.

## Results

### PF-driven slow EPSCs were heterogeneous across cerebellar lobules

A remarkable feature of the cerebellar cortex is its stereotypical anatomy, composed of well-defined regions and lobules that can be reproducibly identified. However, it remains unclear whether Purkinje cells in different cerebellar regions and lobules show uniform or distinct synaptic properties and how this may contribute to region or lobule-specific cerebellar cortex output. To help resolve this, we compared the physiological properties of slow EPSCs at Purkinje cells in lobules located in the midline vermis with those in the laterally positioned floccular lobule (Fig. 1*a*), regions that have different functional roles (Witter and De Zeeuw, 2015a) and molecular profiles (Fujita et al., 2014). We measured PF-driven slow EPSCs using patch-clamp recordings from Purkinje cells (see Materials and Methods) in different lobules of the cerebellum taken from acute mouse brain slices (Fig. 1*b*). The slow EPSC at PF to Purkinje cell synapses was measured in response to physiologically relevant high-frequency PF stimulation in the presence of antagonists of AMPARs and GABA_A_ (γ-amino butyric acid A) receptors (50 µM picrotoxin, 5 µM NBQX; Batchelor and Garthwaite, 1997; Brasnjo and Otis, 2001; Canepari et al., 2004; Chadderton et al., 2004; Jorntell and Ekerot, 2006; van Beugen et al., 2013; Hartmann et al., 2008).

We found that slow EPSCs in vermal lobules IV/V had dynamics surprisingly different from those in the flocculus (Fig. 1*b–d*). In particular, the time of peak of lobules IV/V slow EPSCs was up to threefold lower than the time of peak of floccular slow EPSCs (Fig. 1*d*). Moreover, slow EPSCs in lobules IV/V were less variable than those in the flocculus (Fig. 1*d,e*). Next, we measured slow EPSCs from lobule X of the vermis. Lobule X is similar to the flocculus in terms of being largely zebrin-positive, in contrast to the largely zebrin-negative lobules IV/V. We found that the times of peak of slow EPSCs in lobule X were similar to those in the flocculus and more delayed than those in lobules IV/V (Fig. 1*d*). Purkinje cells in lobule X, like those in the flocculus, displayed heterogeneous timing of their slow current events (Fig. 1*d,e*). The heterogeneity we observed in slow EPSC dynamics was also reflected by slower decay times and longer half-widths in vermal lobule X and the flocculus, compared with vermal lobules IV/V (Fig. 1*f,g*). In addition, the slow EPSC had a slightly lower amplitude in lobule X, which aligned with previous findings (Fig. 1*c*; Wadiche and Jahr, 2005). Next, we measured how the size of the slow EPSC impacts its timing by comparing the peak amplitude versus the time of peak of each cell across the three lobules. Regardless of the peak amplitude of the slow EPSC in lobules IV/V, the time of peak had a low variance when compared with other lobules (Fig. 1*h*, pink). In marked contrast, slow EPSCs from the flocculus (Fig. 1*h*, blue) formed a nonoverlapping population with a delayed time of peak. Finally, slow EPSCs from lobule X (Fig. 1*h*, green) showed a time of peak in between these two populations but were distinguished by their longer decay times. Thus, Purkinje cells possess distinct timing and shape of slow EPSCs depending on their location in different lobules.

In contrast to the differences observed with the slow EPSCs, we could not detect a difference in the timing of fast EPSCs in lobules IV/V and the flocculus (Extended Data Fig. 1-1). Thus, the timing heterogeneity in slow EPSCs was specific to this current and was not a feature of the synapse or the cell.

### Zebrin identity did not determine slow EPSC properties

The differences in slow EPSCs in lobules IV/V and the flocculus could be due to a variety of molecular and cellular signatures of Purkinje cells in these lobules. One molecular signature that distinguishes Purkinje cells with different properties is zebrin. It was previously shown that Purkinje cells in vermal lobules IV/V are largely zebrin-negative while those in vermal lobule X and the flocculus are largely zebrin-positive (Fujita et al., 2012; Fig. 2*a–d*). However, some Purkinje cells within these lobules do not follow the same zebrin identity as the majority of other Purkinje cells within the lobule. We took advantage of this intrinsic variability to interrogate how the zebrin identity of individual Purkinje cells relates to their slow EPSC timing. In order to do this, patch-clamp recordings from different regions were first performed, during which the recorded cell was dye-filled. Slices were subsequently fixed and immunolabelled retrospectively for zebrin (Fig. 2*e,f*). This way, the zebrin identities of individual cells were identified after characterizing their slow EPSCs. As expected, most cells in each region followed the zebrin identity of that lobule. However, as expected, instances were found where Purkinje cells had the opposite zebrin identity (Fig. 2*g*). Surprisingly, we found that the identity of the lobule rather than an individual Purkinje cell's zebrin identity defined the timing of the slow EPSC (Fig. 2*g*). For example, the sparse zebrin-positive Purkinje cells in lobules IV/V had slow EPSC currents that followed the relatively fast kinetics of the majority zebrin-negative cells within that region. A mirror effect was seen for Purkinje cells in the flocculus and lobule X, where sparse zebrin-negative cells showed slow EPSCs with kinetics similar to Purkinje cells that were zebrin-positive. Thus, our results show that cerebellar lobule rather than individual Purkinje cell zebrin expression determined slow EPSC properties.

### Slow EPSC heterogeneity persisted across a range of parameters

**Figure 4.**
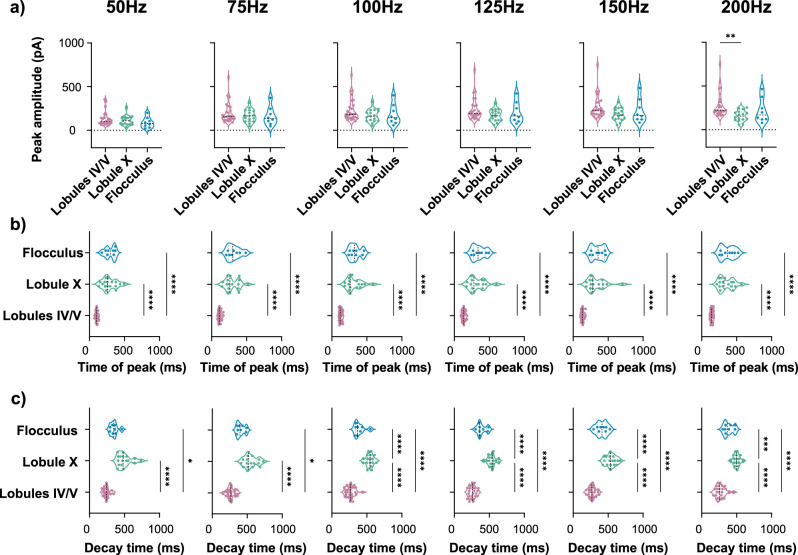
Heterogeneity of sEPSC properties persisted across a range of PF stimulation frequencies. ***a***, From left to right, Peak amplitude of sEPCS in lobules IV/V, lobule X, and the flocculus are similar, in response to 10 PF stimulations at 50, 75, 100, 125, 150, and 200 Hz. **p < 0.01, Kruskal–Wallis test followed by Dunn's multiple-comparisons test. ***b***, From left to right, sEPSC time of peak is earlier in lobules IV/V in comparison with lobule X and the flocculus, in response to 10 PF stimulations at 50, 75, 100, 125, 150, and 200 Hz. ****p < 0.0001, Kruskal–Wallis test followed by Dunn's multiple-comparisons test. ***c***, From left to right, Time for the sEPSC peak amplitude to decay from 90 to 10% was longer for both lobule X and the flocculus in comparison with lobules IV/V, in response to 10 PF stimulations at 50, 75, 100, 125, 150, and 200 Hz. 50 Hz, 75 Hz *p < 0.05, ****p < 0.0001, Kruskal–Wallis test followed by Dunn's multiple-comparisons test. 100–200 Hz ***p < 0.001, ****p < 0.0001, ordinary one-way ANOVA followed by Tukey's multiple-comparisons test. In order to compare the effect of PF stimulation frequency on the sEPSC, the 100 Hz dataset included a subset of the cells that contributed to Figure 1, as well as to Figure 9. Violin plots show median and quartiles. (50 Hz: lobules IV/V = 24 cells from 18 mice, lobule X = 17 cells from 17 mice, flocculus = 8 cells from 6 mice; 75 Hz: lobules IV/V = 26 cells from 18 mice, lobule X = 17 cells from 17 mice, flocculus = 8 cells from 6 mice; 100 Hz: lobules IV/V = 28 cells from 18 mice, lobule X = 17 cells from 17 mice, flocculus = 8 cells from 6 mice; 125 Hz: lobules IV/V = 25 cells from 18 mice, lobule X = 17 cells from 17 mice, flocculus = 8 cells from 6 mice; 150 Hz: lobules IV/V = 25 cells from 17 mice, lobule X = 17 cells from 17 mice, flocculus = 8 cells from 6 mice; 200 Hz: lobules IV/V = 24 cells from 16 mice, lobule X = 16 cells from 16 mice, flocculus = 8 cells from 6 mice). Additional details on statistics are in Extended Data Table 1.

It is possible that the heterogeneity we observed is only present with the specific PF stimulation parameters we used (10 pulses at 100 Hz), which would limit its relevance. We ruled out this possibility by testing a range of parameters. We found that the heterogeneity in slow EPSCs remained even with a lower number of stimuli [starting with three pulses, shown to be the lowest number to activate mGluR1 (Brasnjo and Otis, 2001), and going up to five stimuli; Fig. 3*a–i*]. In addition, we maintained the number of stimuli at 10 but tested a range of stimulation frequencies, from 50 to 200 Hz. Across all frequencies, the time of peak of the slow EPSC in lobules IV/V was early and highly homogeneous, in contrast to the delayed and varied time of peak in lobule X and the flocculus. In all cases, the decay was different across lobules in a manner similar to that described in Figure 1 (Fig. 4*a–c*). Thus, the specific number of stimuli or the frequency did not determine the heterogeneity in slow EPSCs.

We were concerned that the time after break-in for whole-cell patching could contribute to dialysis of the intracellular cytosol and impact pathways necessary for the slow EPSC (Sakmann and Neher, 1984; Rae and Fernandez, 1988; Cahalan and Neher, 1992). To account for this, we analyzed a subset of Purkinje cells from Figure 1 in which slow EPSCs were measured within the restricted time window of 10–20 min after break-in. The results showed that even within this restricted subset of cells, the heterogeneity observed across lobules remained (Extended Data Fig. 1-2).

Recent studies describe sex-based differences in cerebellar structure and physiology (Mercer et al., 2016; Steele and Chakravarty, 2018). To test this possibility, we compared slow EPSCs from male and female mice. We found no difference between the slow EPSC properties of Purkinje cells from male and female mice (Extended Data Fig. 1-3).

### Heterogeneity in mGluR1-TRPC3 signaling may contribute to Purkinje cell slow EPSCs

**Figure 5.**
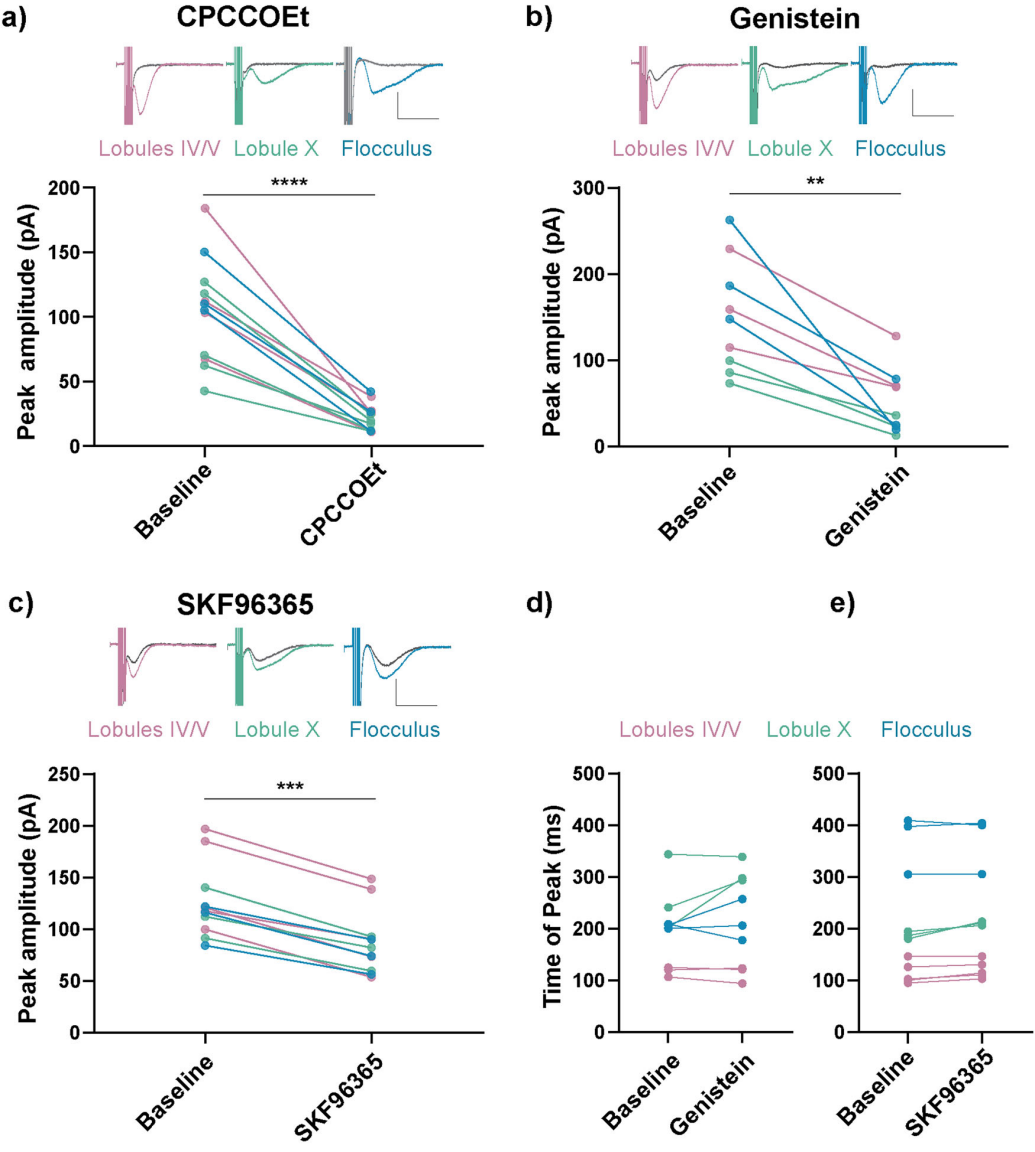
sEPSCs exhibited equivalent mGluR and TRPC3 dependence across lobules. ***a***, Top, Representative sEPSC traces from lobules IV/V, lobule X, and the flocculus. Colored traces are sEPSCs at baseline, and gray traces represent sEPSCs after blockade by wash-in of noncompetitive mGluR1 antagonist CPCCOEt (100 µM). Bottom, sEPSCs were blocked by wash-in of CPCCOEt. ****p < 0.0001, paired *t* test. ***b***, ***c***, Top, Representative sEPSC traces from lobules IV/V, lobule X, and the flocculus. Colored traces are sEPSCs at baseline, and gray traces are sEPSCs after wash-in. Bottom, Wash-in of (***b***) genistein (100 µM) or (***c***) SKF96365 (10 µM) reduced sEPSC amplitude across all cerebellar lobules tested. **p < 0.01, ***p < 0.001, Wilcoxon matched-pairs signed rank test. ***d***, ***e***, Partial blockade of TRPC3 did not explain heterogeneity in time of peak across lobules. Statistics were not run to compare different lobules as the number of cells in each lobule was too low. Scale bar, 100 pA, 0.5 s. Violin plots show median and quartiles. (***a***: lobules IV/V = 4 cells from 3 mice, lobule X = 5 cells from 3 mice, flocculus = 3 cells from 2 mice. ***b***, ***d***: lobules IV/V = 3 cells from 2 mice, lobule X = 3 cells from 2 mice, flocculus = 3 cells from 3 mice. ***c***, ***e***: lobules IV/V = 5 cells from 4 mice, lobule X = 3 cells from 3 mice, flocculus = 3 cells from 3 mice). Additional details on Statistics are in Extended Data Table 1.

The slow EPSC was previously shown to depend on mGluR1 (Hartmann et al., 2008). We wondered if the heterogeneity of the slow EPSC that we found relied on other types of signaling across the different lobules. However, we found that the noncompetitive mGluR1 antagonist CPCCOEt (100 µM; Hartmann et al., 2008) blocked slow EPSCs in all three regions (Fig. 5*a*), arguing against this possibility.

Downstream of mGluR1, the slow EPSC is mediated by the nonselective cation channel TRPC3 (Hartmann et al., 2008; Chae et al., 2012; Cole and Becker, 2023). To test how TRPC3 blockade impacts the time of peak, we used the tyrosine kinase inhibitor genistein (100 µM; Vazquez et al., 2004; Y. Kim et al., 2012b) and the TRPC channel blocker SKF96365 (10 µM; Chae et al., 2012; Song et al., 2014; Cole and Becker, 2023), which both partially block the slow EPSC, allowing us to measure the kinetics of the remaining current. We found that the magnitude of the slow EPSC was consistently reduced by genistein and by SKF96365 across lobules, but there was no shift in timing that could explain the differences between lobules (Fig. 5*b–e*).

**Figure 6.**
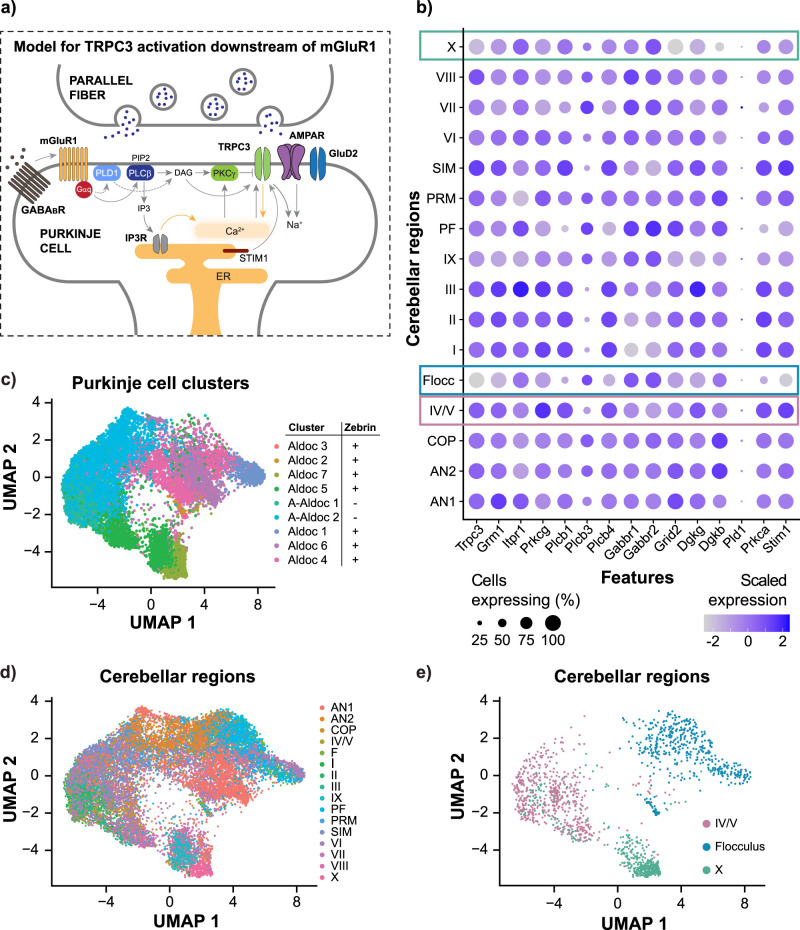
Heterogeneity in mGluR1-TRPC3 signaling, and in overall gene expression, in lobules IV/V, lobule X, and the flocculus. ***a***, Schematic of the postsynaptic signaling pathway involved in the slow EPSC at PF to Purkinje cell synapses (combined and adapted from Hartmann et al., 2011; Kano and Watanabe, 2017; Hirai and Kano, 2018; Cole and Becker, 2023). ***b***, Visualization of gene expression heterogeneity in single-nucleus RNA-seq data of 16,634 Purkinje cells, from Kozareva et al. (2021). The dot plot shows the scaled expression of selected genes of interest across cerebellar regions. The regions of interest are highlighted with appropriately colored boxes (Flocc = flocculus). The following transcripts code for the proteins of interest in ***b***: Trpc3, TRPC3; Grm1, mGluR1; Itpr1, IP_3_ receptor; Prkcg, PKCγ; Plcb, isoforms of PLCβ; Gabbr, GABA_B_ receptor; Grid2, GluD2 receptor; Dgkg, DAG kinase isoforms; Pld1, phospholipase D1; Prkca, protein kinase C; Stim1, stromal interaction molecule 1. ***c***, Visualization of gene expression heterogeneity in scRNA-seq data of 16,634 Purkinje cells as described by Kozareva et al. (2021). UMAP dimension reduction showing original authors’ identified PC clusters. Aldoc and zebrin are equivalent. ***d***, UMAP plot showing the region of the cerebellum that the cells were sampled from, demonstrating the lack of full alignment between molecularly defined cell-type and lobular identity. ***e***, Lobules IV/V, lobule X, and the flocculus are separated out from ***d***, demonstrating that all three regions fall in different clusters, with some overlap. Also see Extended Data Figure 6-1.

The characteristically prolonged time course of the slow EPSC is related to the associated G-protein–dependent signaling cascade (see Fig. 6*a* for a noncomprehensive schematic; Hartmann et al., 2011; Kano and Watanabe, 2017; Hirai and Kano, 2018; Cole and Becker, 2023). However, the signaling pathways connecting mGluR1 and TRPC3 signaling, and its regulation, are not fully understood (Cole and Becker, 2023). Despite this, we wanted to identify candidate molecular players underlying the diversity of slow EPSCs across lobules. Therefore, we performed a bioinformatic analysis of Purkinje cell transcriptomes using a published single-nucleus RNA sequencing dataset (Kozareva et al., 2021). We separated Purkinje cell transcriptomes by lobule of interest and focused on molecular candidates related to the mGluR1-TRPC3 pathway (Fig. 6*b*; Hartmann et al., 2011; Kano and Watanabe, 2017; Hirai and Kano, 2018; Cole and Becker, 2023). Comparison of gene expression demonstrated statistically significant differences across lobules. Key features of our analysis aligned with previous findings. For instance, the expression of Grm1, which encodes mGluR1, was greater in lobules IV/V than in the flocculus and lobule X. This is consistent with greater mGluR1b in zebrin-negative zones (Mateos et al., 2001). TRPC3 expression was also greater in the largely zebrin-negative lobules IV/V, in contrast to the largely zebrin-positive flocculus and lobule X. This is consistent with previous studies, although it is also known that TRPC3 expression does not perfectly overlap with zebrin zones (Wu et al., 2019).

10.1523/JNEUROSCI.0455-24.2024.f6-1 Figure 6-1 **Buffering intracellular calcium did not change heterogeneity in synaptic timing.** sEPSC recordings were performed with 10 mM EGTA and 1mM BAPTA added to the internal solution. a) Peak amplitude of the sEPSC did not vary between lobules (b-d) However, heterogeneity in the time of peak, half-width, and decay time of the sEPSC persisted. Statistical comparisons: (a, b, c, d) *p < 0.05, **p < 0.01, Kruskal-Wallis test followed by Dunn’s multiple comparisons test. (Fig 6-1 a-d: Lobules IV/V = 6 cells from 4 mice, lobule X = 5 cells from 3 mice, flocculus = 5 cells from 3 mice) Download Figure 6-1, TIF file.

We then focused our analysis on the molecules that distinguish regions with slow timing (lobule X and flocculus) from regions with fast timing (lobules IV/V). We found that there were several differences in gene expression, including in the following: (1) Prkcg, encoding PKCγ (protein kinase C γ); (2) Plcb1 and 4, encoding PLCβ (phospholipase C-β); (3) Dgkg, encoding DAG kinase γ; and (4) Gabbr2, encoding GABA_B_ (γ-aminobutyric acid type B) receptors. In addition, Grid, encoding GluD2 (glutamate receptor delta) channels, had lower expression in lobule X relative to the other two regions (see Discussion). Finally, stromal interaction molecule STIM1 is a key controller of mGluR1-dependent synaptic transmission and differs between lobules IV/V versus lobule X and the flocculus. However, it is also different between the two regions that both have slow timing, lobule X and the flocculus. Thus, we identified more than one molecular correlate of heterogeneity across lobules, within known components of mGluR1-TRPC3 signaling pathways. In addition, these signaling components are likely to interact with each other (Cole and Becker, 2023). Moreover, our bioinformatic analysis by separating cells from different lobules obscures the within-lobule heterogeneity observed both in previous studies of the Purkinje cell transcriptome (Kozareva et al., 2021) and in our data. Overall, we found several key differences in the signaling pathways that could mediate slow EPSC heterogeneity across lobules.

Analyzing their single-cell RNA-seq dataset of cerebellar Purkinje cells, Kozareva et al. (Kozareva et al., 2021) describe nine molecularly defined clusters that could be separated by zebrin identity: two clusters of zebrin-negative cells and seven clusters of zebrin-positive cells. We examined the transcriptomic profiles of Purkinje cells from the three lobules that we are interested in, in terms of these nine clusters (Fig. 6*c*). Surprisingly, lobular clusters do not fully align with the clusters defined by gene expression pattern (Fig. 6*d,e*), and the three regions we investigated fall into separate clusters. This observation may serve to explain the large amount of heterogeneity that we found across single cells. It also suggests that there is high-dimensional transcriptomic heterogeneity within and across regions of the cerebellum.

Next, we assessed whether and how buffering intracellular calcium might affect the heterogeneity of timing of the slow EPSC. However, heterogeneity in slow EPSC timing persisted in recordings performed with 10 mM EGTA and 1 mM BAPTA in the internal solution, demonstrating that, at least at these concentrations, there is no effect on the heterogeneity in timing (Extended Data Fig. 6-1).

**Figure 7.**
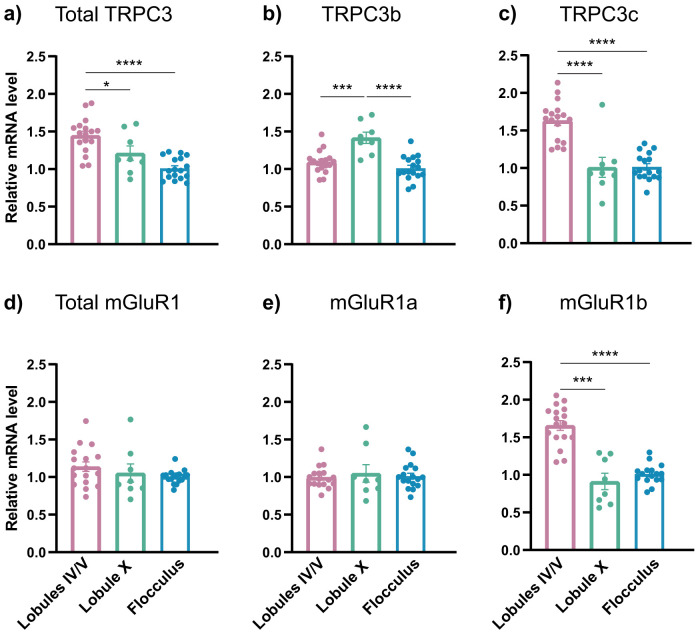
TRPC3 and mGluR1 isoform expression was heterogeneous across cerebellar lobules. Relative mRNA levels of (***a***) total TRPC3, (***b***) TRPC3b isoform, and (***c***) TRPC3c isoform were heterogeneous across lobules IV/V, lobule X, and the flocculus. Relative mRNA level (***d***) total mGluR1, (***e***) mGluR1a, and (***f***) mGluR1b across the three lobules demonstrate heterogeneity in mGluR1b expression. ***a–c***, *p < 0.05, ***p < 0.001, ****p < 0.0001, ordinary one-way ANOVA followed by Tukey's multiple-comparisons test. ***d–f***, ***p < 0.001, ****p < 0.0001, Kruskal–Wallis test followed by Dunn's multiple-comparisons test. All data are mean ± SEM. Additional details on statistics are in Extended Data Table 1.

While the heterogeneity in the signaling pathway connecting mGluR1 to TRPC3 can explain the heterogeneity in slow EPSC we observe, it has also been shown that TRPC3 itself has different splice variants (Y. Kim et al., 2012b). The distribution of splice variants across lobules in the cerebellum has not, to our knowledge, been previously investigated. Therefore, in order to further investigate the splice variants of the receptor and channel mediating the slow EPSC, we performed quantitative reverse transcription-PCR analysis. We separated lobules IV/V and X of the vermis, as well as the flocculus, extracted RNA, and tested the relative abundance of mGluR1, TRPC3, and their splice variants (Fig. 7*a–f*). Lobules IV/V showed higher expression levels of mGluR1b when compared with lobule X and the flocculus, which agrees with published differences in the isoforms of mGluR1 across zebrin-positive versus negative regions (Mateos et al., 2001). Surprisingly, we also found a difference between the expression of the splice variants of TRPC3, TRPC3c, and 3b (Y. Kim et al., 2012b). TRPC3c showed increased expression in lobules IV/V, the region with fast kinetics, compared with lobule X and the flocculus. In agreement with previous literature showing an anticorrelation between the expression levels of TRPC3 and zebrin across lobules (Wu et al., 2019), the expression of total TRPC3 was also higher in lobules IV/V. Taken together, our results identified a correlation between the expression levels of the splice variants TRPC3c and mGluR1b and shorter versus longer dynamics of the slow EPSC.

**Figure 8.**
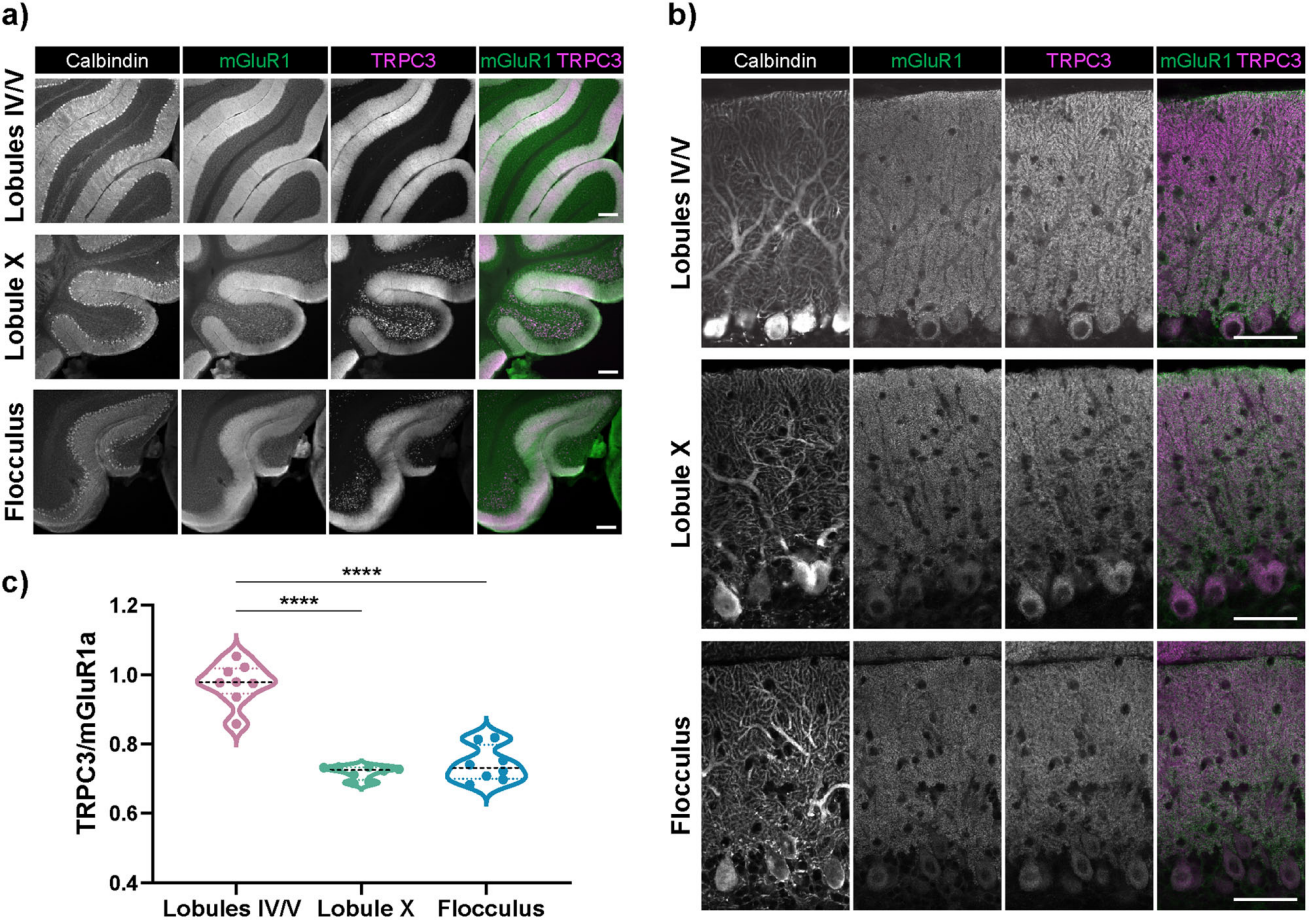
TRPC3 to mGluR1a ratio was higher in lobules IV/V, but mGluR1 and TRPC3 localization was the same across lobules. ***a***, ***b***, Antibody staining for calbindin, mGluR1a, and TRPC3 on 50-µm-thick cerebellar sections demonstrates similar cellular localization across lobules. Scale bars: ***a***, 100 µm; ***b***, 50 µm. ***c***, Mean fluorescence intensities of TRPC3 and mGluR1a were measured in the molecular layer of each lobule, the background intensity was subtracted, and the ratio of TRPC3 to mGluR1 was determined. Each data point is one region from one brain section. For each region (lobules IV/V, lobule X, flocculus), there are two sections per mouse and four mice per region. One-way ANOVA followed by Tukey's multiple-comparisons test, ****p < 0.0001.

Previously, it has been shown that mGluR1 and TRPC3 are localized in the molecular layer, where the Purkinje cell dendritic tree and parallel fiber synapses are (Grandes et al., 1994; Wu et al., 2019). In order to confirm that this is true across the lobules we analyzed, mGluR1a [which is distributed through all lobules (Grandes et al., 1994; Yamasaki et al., 2021)] and TRPC3 were visualized across lobules using antibody staining. As expected, immunofluorescence signals for both mGluR1 and TRPC3 were present in Purkinje cells and throughout their dendritic tree in the molecular layer in all three regions (Fig. 8*a,b*). Moreover, the ratio of fluorescence intensity of TRPC3 to mGluR1a was higher in lobules IV/V than in lobule X and the flocculus, which is consistent with the higher TRPC3 seen in zebrin-negative regions (Wu et al., 2019) and with our RT-qPCR data (Fig. 8*c*).

### Slow EPSC heterogeneity diversifies the timing of Purkinje cell firing

**Figure 9.**
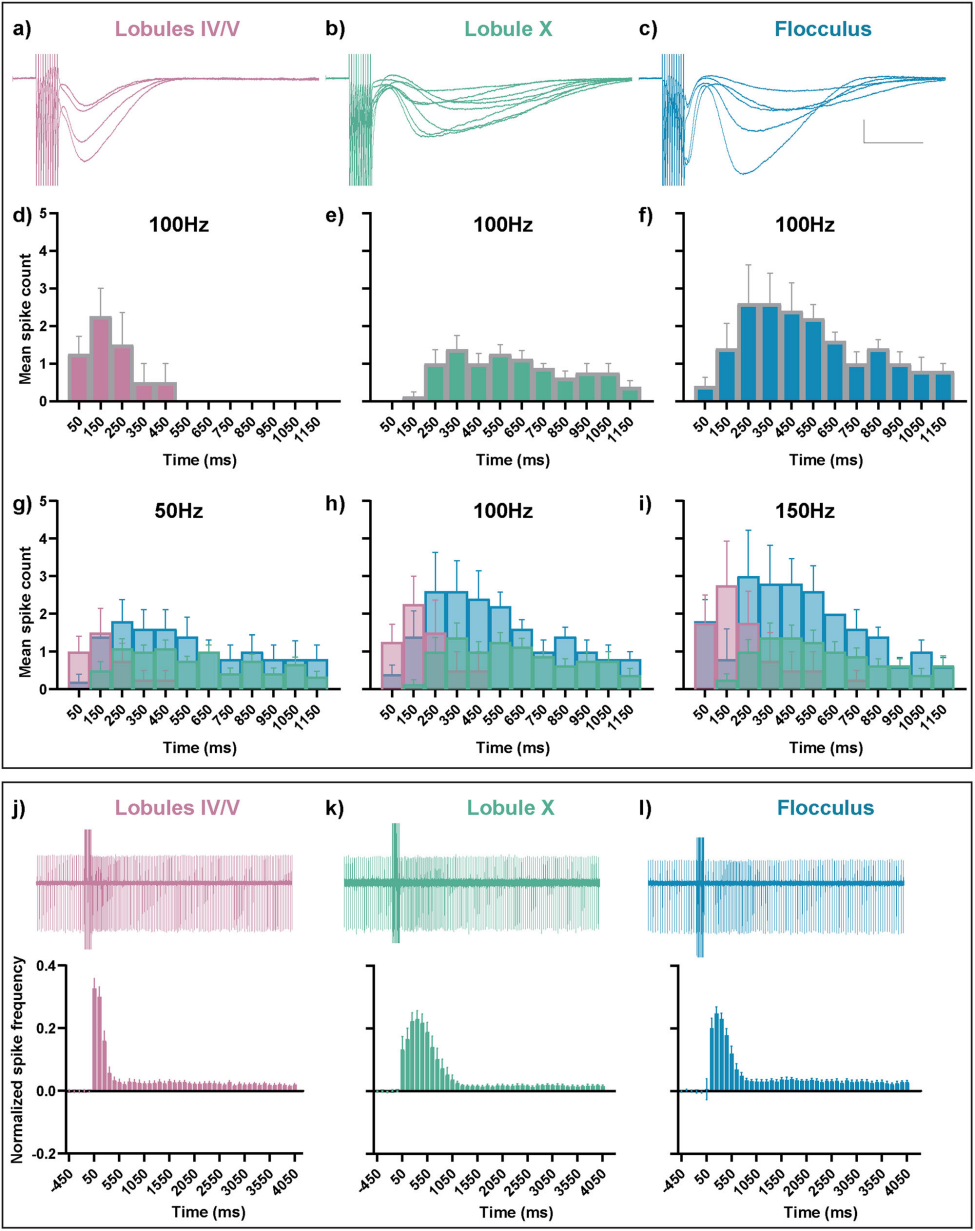
sEPSC heterogeneity diversifies Purkinje cell spike timing. ***a–c***, sEPSC traces in response to 10 PF stimulations at 100 Hz recorded from Purkinje cells voltage-clamped at −70 mV. Scale bar 100 pA, 250 ms. ***d–f***, Mean spike counts binned into 100 ms time bins from the same Purkinje cells, spiking in current-clamp conditions, in response to the same stimulation of 10PF stimulations at 100 Hz. ***g–i***, Overlay of 100 ms time-binned mean spike counts from the same Purkinje cells under current-clamp conditions in response to 10PF stimulations at (***g***) 50 Hz, (***h***) 100 Hz, and (***i***) 150 Hz. ***j***, Top, Representative trace of a cell-attached recording from lobules IV/V, with PF stimulation at time 0 and 10 stimulus pulses at 100 Hz. Bottom, Normalized instantaneous spike frequency binned into 100 ms time bins. The representative cell-attached trace is aligned in time to the *x*-axis of the graph. ***k***, ***l***, Same as in ***j*** but for lobule X and the flocculus, respectively. All data are mean ± SEM. (**a**–**i**: lobules IV/V = 4 cells from 4 mice, lobule X = 8 cells from 8 mice, flocculus = 5 cells from 4 mice; **j**–**l**: lobules IV/V = 11 cells from 4 mice, lobule X = 8 cells from 3 mice, flocculus = 9 cells from 3 mice).

Next, we wondered how the slow EPSC heterogeneity would impact Purkinje cell spiking. It is known that the firing properties of Purkinje cells in response to depolarization are not uniform across the cerebellum (C. H. Kim et al., 2012a; Zhou et al., 2014). To explore this, cells were first recorded in voltage-clamp configuration to measure the slow EPSC (Fig. 9*a–c*). Next, recordings were switched to current-clamp configuration, allowing Purkinje cells to fire action potentials in response to synaptic input (Fig. 9*d–f*). This allowed us to look directly at any relationship between slow EPSC timing and parallel fiber-evoked modulation of spiking. We uncovered a difference in the timing of the firing response across lobules that matched the slow EPSC kinetics. Lobules IV/V, which had faster EPSC kinetics, had an equivalently short firing response. In contrast, slow EPSCs in lobule X and the flocculus, which had a diversity of longer delays, triggered prolonged firing responses in comparison with the responses elicited in lobules IV/V (Fig. 9*d–i*).

We were concerned that the potentially dialyzing effects of whole-cell recordings (Sakmann and Neher, 1984; Rae and Fernandez, 1988; Cahalan and Neher, 1992) affected our results. We also wondered what the effect of slow EPSC heterogeneity was on intrinsically firing cells. To address these concerns, we explored how slow EPSC heterogeneity affected Purkinje cell firing using cell-attached recordings. To isolate slow EPSC-driven effects, both AMPARs and GABA_A_ receptors were blocked with 5 µM NBQX and 50 µM picrotoxin, as previously. We observed that different regions of the cerebellum showed heterogeneity in the timing of their firing response (Fig. 9*j–l*), which validated our previous whole-cell recordings (Fig. 9*a–i*). The enhancement of firing was brief in lobules IV/V, in comparison with the delayed and prolonged enhancement of firing in lobule X and the flocculus.

**Figure 10.**
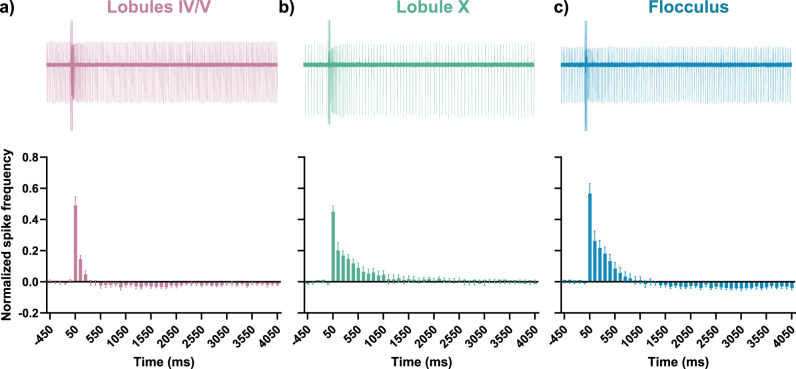
There is heterogeneity in parallel fiber-driven Purkinje cell firing even when fast synaptic transmission was not blocked. ***a–c***, Top, Representative trace of cell-attached recordings, where 0 marks the time of five PF stimulations at 100 Hz. Bottom, Normalized spike frequency binned into 100 ms bins for (***a***) lobules IV/V, (***b***) lobule X, and (***c***) the flocculus. These recordings did not contain NBQX or picrotoxin, and therefore fast excitatory and inhibitory synaptic transmission were intact. (Lobules IV/V = 13 cells from 5 mice, lobule X = 9 cells from 4 mice, flocculus = 10 cells from 3 mice).

An important limitation of these data is that GABAergic inhibition is blocked, although fast inhibitory synaptic transmission is a major determinant of Purkinje cell firing. In addition, AMPAR-dependent fast synaptic transmission is also blocked, in order to visualize the time course of the mGluR1-dependent slow synaptic response. Therefore, in order to identify whether the heterogeneous responses to parallel fiber input across lobules hold true even in the presence of GABAergic inhibition and fast glutamatergic transmission, we performed cell-attached recordings in the absence of picrotoxin and NBQX. Cell-attached recordings were made from Purkinje cells in each of the three regions, lobules IV/V, lobule X, and the flocculus, while parallel fibers were stimulated five times at 100 Hz to trigger mGluR1-dependent slow synaptic currents (Fig. 10*a–c*). As expected, the absence of a fast synaptic transmission block resulted in a more rapid response to stimuli across all three lobules. In addition, the absence of an inhibition block led to a more rapid return to baseline firing rate. However, the marked differences in response timings across the three lobules were still observed, with Purkinje cells in lobules IV/V showing a relatively short increase in firing rate compared with cells in lobule X and the flocculus (Fig. 10*a–c*). Thus, the heterogeneity in Purkinje cell responses remains true even under conditions where GABAergic inhibition and fast glutamatergic transmission are intact.

Taken together, our findings demonstrate that the same synaptic input led to an increase in firing rate over different timescales across lobules. Thus, the same synaptic input diversified PF-triggered responses heterogeneously across the cerebellum.

## Discussion

Our study reveals how Purkinje cells in different locations of the cerebellum transform the same synaptic input into heterogeneous outputs that vary in timing and dynamics. The difference in slow EPSC timing across lobules of the cerebellum correlated well with components of the mGluR1-TRPC3 signaling pathway, implicating multiple molecular players in the regulation of slow synaptic signaling. Our results highlight a hitherto unappreciated form of cellular heterogeneity that complements and expands the known repertoire of cerebellar diversity (Cerminara et al., 2015).

### Anatomical and molecular subdivisions of the cerebellum

Architecturally, the cortex of the cerebellum consists of five transverse zones (Apps and Hawkes, 2009) and three longitudinal compartments, containing the folded lobules. Although individual lobules have been correlated with specific functions, the fissures between lobules do not necessarily demarcate functionally distinct circuits (Apps and Hawkes, 2009; Witter and De Zeeuw, 2015a). Functionally, the circuit between the inferior olive, the cerebellar cortex, and the deep cerebellar nuclei (and vestibular nucleus) is demarcated longitudinally into modules. Modules are thus defined by their connectivity and are further subdivided into microzones, which receive common climbing fiber input (reviewed by Apps et al., 2018). Another way to compartmentalize the cerebellar cortex consists of the parasagittal bands or stripes that demarcate differences in Purkinje cell molecular properties and information processing, most commonly described by zebrin identity (De Zeeuw, 2021). Of note, the zebrin identity of a parasagittal band of Purkinje cells correlates or anticorrelates with 126 different molecules (Rodriques et al., 2019), including signaling components of the mGluR1 pathway. Since both mGluR1 and TRPC3 are critical for cerebellar synapses to function normally and to undergo plasticity (Aiba et al., 1994; Yamakawa and Hirano, 1999; Ichise et al., 2000; Kishimoto et al., 2002; Knöpfel and Grandes, 2002; Hartmann et al., 2004, 2008; Lüscher and Huber, 2010; Ohtani et al., 2014; Hartmann and Konnerth, 2015; Kano and Watanabe, 2017), the parasagittal location of a Purkinje cell has implications for how it processes information and how synapses onto it undergo long-term potentiation or LTD. It is also important to note that the alignment of mGluR1 signaling–related molecules and zebrin is not always exact. Generally, mGluR1b is present in complementary bands to zebrin (Mateos et al., 2001); the IP_3_ receptor, which can modulate TRPC3 (Y. Kim et al., 2012b), is present in bands similar to zebrin, although not exactly overlapping (Furutama et al., 2010). PLCβ3 and PLCβ4 are expressed in mostly nonoverlapping bands, with PLCβ3 expression in zebrin-positive PCs, depending on location within the anteroposterior transverse zone, and PLCβ4 expression in zebrin-negative PCs (Sarna et al., 2006). TRPC3 itself is expressed in a roughly complementary fashion to zebrin (Wu et al., 2019). All of these molecules directly impact both baseline transmission and plasticity at parallel fiber synapses. However, individual parallel fibers can extend across parasagittal Purkinje cell stripes (Apps et al., 2018), thereby carrying the same information to different microzones.

### The role of zebrin

We assessed the relationship of our observations of Purkinje cell heterogeneity in light of zebrin-positive or zebrin-negative identity. However, as described above, rather than having just two possible types of molecular signature, Purkinje cells have diverse molecular properties (Guo et al., 2021; Kozareva et al., 2021). In addition, zebrin expression is also not necessarily binary, i.e., solely zebrin-positive or zebrin-negative but has been described as having graded expression (Fujita et al., 2014). A broad diversity of cellular properties would serve to expand the computational capacity of the cerebellum. Our analysis of Purkinje cell transcriptomes also supports this idea of diversity in multiple different interacting molecules.

As a consequence of focusing on the slow EPSC, our study highlights the mGluR1 pathway. mGluR1 and its downstream signaling cascade are essential for normal cerebellar function, mutations in mGluR1 impair motor coordination, cerebellum-dependent learning, and proper circuit development (Aiba et al., 1994; Ichise et al., 2000; Ohtani et al., 2014). Both mGluR1 and TRPC3 are critical for normal cerebellar function (Yamakawa and Hirano, 1999; Kishimoto et al., 2002; Knöpfel and Grandes, 2002; Hartmann et al., 2004, 2008; Lüscher and Huber, 2010; Hartmann and Konnerth, 2015; Kano and Watanabe, 2017). They are also important to understand as therapeutic targets because of their dysfunction in cerebellum-dependent diseases (Knöpfel and Grandes, 2002; Becker et al., 2009; Lüscher and Huber, 2010; Becker, 2014; Dulneva et al., 2015; Meera et al., 2017; Tiapko and Groschner, 2018; Crupi et al., 2019; Cole and Becker, 2023). Zebrin was potentially a relevant marker of heterogeneity for our study because of zebrin-based patterning of components of the mGluR-signaling pathway (Mateos et al., 2001; Wadiche and Jahr, 2005; Sarna et al., 2006; Furutama et al., 2010; Wu et al., 2019). However, our data demonstrated that zebrin identity alone did not determine slow EPSC timing.

### Key molecular determinants of synaptic diversity

Our analysis of published Purkinje cell transcriptomes to investigate regional differences highlights several molecules that are differentially expressed in the lobules with different slow EPSC dynamics. There is an increase in Prkcg expression, which encodes PKCγ, in the region with faster slow EPSCs relative to the other two regions. As summarized in Figure 6*a*, PKCγ negatively regulates TRPC3, which would be consistent with a shorter or more tightly timed slow EPSC (Hartmann et al., 2011; Cole and Becker, 2023). Plcb1, 3, and 4, encoding PLCβ (Hashimoto et al., 2001; Miyata et al., 2001), are also differentially expressed in lobules IV/V versus lobule X and the flocculus; lobule X and the flocculus also have different expression levels. Potentially, this could lead to faster activation of TRPC3 in lobules IV/V. In addition, Dgkg, encoding DAG kinase γ, is higher in lobules IV/V than in the other two regions. This is particularly interesting, as DAG-kinase has been shown to directly control the response duration of mGluR1 (Guo et al., 2021), which is consistent with our results. Grid2, encoding GluD2 channels, had a lower expression in lobule X relative to the other two regions. Since loss of GluD2 regulates mGluR1-TRPC3 signaling (Kato et al., 2012; Ady et al., 2014), this may contribute to the characteristically small-amplitude and long-decay sEPSCs in lobule X. Our analysis also showed that Gabbr2, encoding GABA_B_ receptors, was expressed at a higher level in the regions with slow dynamics, i.e., lobule X and the flocculus, relative to the region with fast dynamics, lobules IV/V. GABA_B_ receptors have been implicated in mGluR1-TRPC3 interactions and in the mGluR1-dependent slow EPSC, although the mechanisms by which it does so remain unknown (Tian and Zhu, 2018). Our comparison of lobular identity with previously described, transcriptionally defined categories of Purkinje cells also suggests a diversity of categories within a lobule (Fig. 6; Kozareva et al., 2021). Moreover, Kozareva et al. described greater heterogeneity in zebrin-positive clusters of Purkinje cells (Kozareva et al., 2021), an observation that correlates with the greater heterogeneity in slow EPSC properties that we see in the largely zebrin-positive lobule X and flocculus, in comparison with the relatively uniform slow EPSC properties in lobules IV/V. Our findings are also consistent with a recent study (Guo et al., 2021), which demonstrated diversification of timing in a cell-autonomous manner by mGluR1 signaling, in the context of cerebellar unipolar brush cells.

### Heterogeneity of TRPC3 isoforms

Our results also demonstrated a correlation between increased expression of the splice variant TRPC3c and shorter slow EPSCs in lobules IV/V. TRPC3c is distinguished from TRPC3b by the loss of a single exon, which codes for a large part of the calmodulin-IP_3_ receptor binding domain (Y. Kim et al., 2012b). Thus, it has altered intracellular regulation and sensitivity to Ca^2+^. In addition, there is an increased channel opening rate. However, the regulation and function of TRPC3 splice variants in Purkinje cells in vivo is not well understood, and it remains unclear how TRPC3c alone can directly cause faster sEPSCs (Hartmann et al., 2011; Nelson and Glitsch, 2012; Y. Kim et al., 2012b; Hartmann and Konnerth, 2015; Sierra-Valdez et al., 2018). Indeed, our results also demonstrate that there are additional molecular players that are likely to be responsible for different shapes and timing of the slow EPSC. Overall, our findings point to the necessity to more fully understand the expression and regulation of the mGluR1-TRPC3 cascade (Bodzęta et al., 2021; Cole and Becker, 2023) and highlight that previously undescribed regional heterogeneity in splice variants needs to be considered.

### Implications for plasticity

Both mGluR1 and TRPC3 are essential for LTD at PF to Purkinje cell synapses (Aiba et al., 1994; Chae et al., 2012; S. J. Kim, 2013),and LTD is critical for behavioral learning (Suvrathan et al., 2016; Suvrathan and Raymond, 2018; De Zeeuw et al., 2021). However, LTD is directly dependent on the postsynaptic Ca^2+^ signal (Wang et al., 2000; Ito, 2001). The slow EPSC and the Ca^2+^ signal are thought to be two independent intracellular signaling cascades downstream of mGluR1 (Takechi et al., 1998; Hartmann et al., 2011; Hartmann and Konnerth, 2015; Ouares and Canepari, 2020). Therefore, the timing of the slow EPSC is likely not related to the timing requirements for plasticity. However, it remains unclear how the diverse response timings of TRPC3 interact with heterogeneous plasticity mechanisms across the cerebellum, such as the demarcation into upbound and downbound zones (De Zeeuw, 2021).

### The link to Purkinje cell firing patterns

The slow EPSC-driven heterogeneity in firing we describe here adds to previous descriptions of differences in firing rate between Purkinje cells in zebrin-positive and zebrin-negative regions (Zhou et al., 2014), and the diverse intrinsic input–output relationships in different lobules (C. H. Kim et al., 2012a). It was previously known that mGluR1 blockade can reduce Purkinje cell firing frequency (Yamakawa and Hirano, 1999) and TRPC3 perturbation can affect Purkinje cell firing rate (Sekerková et al., 2013) in a zebrin-dependent manner (Zhou et al., 2014), although the underlying mechanisms remain unclear.

The high-frequency spontaneous firing of Purkinje cells encodes the output of the cerebellar cortex and provides tonic inhibition to downstream cells in the deep cerebellar and vestibular nuclei. Consequently, the impact of homogeneous or diversified slow current timings, such as we observe in lobules IV/V versus lobule X and the flocculus, is that it could affect the synchrony or lack thereof of Purkinje cells that are activated by the same PF beam. As a result, time-locked spiking of downstream deep cerebellar nucleus (DCN) neurons may be affected, as well as a direct impact on the firing rate of DCN neurons (Telgkamp and Raman, 2002; Person and Raman, 2012; Sedaghat-Nejad et al., 2022).

### Future directions

Our results highlight the importance of synaptic heterogeneity in Purkinje cell timing, which opens new areas of inquiry into how cerebellar circuits utilize this diversity. Our findings also expand the repertoire of synaptic and cellular mechanisms that different regions of the cerebellum may draw on to support diverse functions. Finally, although we did not causally link mGluR1-TRPC3 signaling to this diversity, we reveal tight and suggestive correlations with key molecular players, which provides a firm foundation for future mechanistic studies into the role of synaptic heterogeneity in Purkinje cell timing. Thus, our study is not a final verdict on Purkinje cell timing diversity but rather a key first step.

## References

[B1] Ady V, Perroy J, Tricoire L, Piochon C, Dadak S, Chen X, Dusart I, Fagni L, Lambolez B, Levenes C (2014) Type 1 metabotropic glutamate receptors (mGlu1) trigger the gating of GluD2 delta glutamate receptors. EMBO Rep 15:103–109. 10.1002/embr.201337371 24357660 PMC4303454

[B2] Aiba A, Kano M, Chen C, Stanton ME, Fox GD, Herrup K, Zwingman TA, Tonegawa S (1994) Deficient cerebellar long-term depression and impaired motor learning in mGluR1 mutant mice. Cell 79:377–388. 10.1016/0092-8674(94)90204-6 7954803

[B3] Apps R, et al. (2018) Cerebellar modules and their role as operational cerebellar processing units: a consensus paper. The Cerebellum 17:683–684. 10.1007/s12311-018-0959-9 29931663 PMC6828140

[B4] Apps R, Hawkes R (2009) Cerebellar cortical organization: a one-map hypothesis. Nat Rev Neurosci 10:670–681. 10.1038/nrn2698 19693030

[B5] Batchelor AM, Garthwaite J (1997) Frequency detection and temporally dispersed synaptic signal association through a metabotropic glutamate receptor pathway. Nature 385:74–77. 10.1038/385074a0 8985249

[B6] Batchelor AM, Madge DJ, Garthwaite J (1994) Synaptic activation of metabotropic glutamate receptors in the parallel fibre-Purkinje cell pathway in rat cerebellar slices. Neuroscience 63:911–915. 10.1016/0306-4522(94)90558-4 7535396

[B7] Becker EBE (2014) The moonwalker mouse: new insights into TRPC3 function, cerebellar development, and ataxia. Cerebellum 13:628–636. 10.1007/s12311-014-0564-5 24797279 PMC4155175

[B8] Becker EBE, Oliver PL, Glitsch MD, Banks GT, Achillic F, Hardy A, Nolan PM, Fisher EMC, Davies KE (2009) A point mutation in TRPC3 causes abnormal Purkinje cell development and cerebellar ataxia in moonwalker mice. Proc Natl Acad Sci U S A 106:6706–6711. 10.1073/pnas.0810599106 19351902 PMC2666615

[B9] Bodzęta A, Scheefhals N, MacGillavry HD (2021) Membrane trafficking and positioning of mGluRs at presynaptic and postsynaptic sites of excitatory synapses. Neuropharmacology 200:108799. 10.1016/j.neuropharm.2021.108799 34592242

[B10] Bosman LWJ, et al. (2010) Encoding of whisker input by cerebellar Purkinje cells. J Physiol 588:3757–3783. 10.1113/jphysiol.2010.195180 20724365 PMC2998225

[B11] Brasnjo G, Otis TS (2001) Neuronal glutamate transporters control activation of postsynaptic metabotropic glutamate receptors and influence cerebellar long-term depression. Neuron 31:607–616. 10.1016/S0896-6273(01)00377-4 11545719

[B12] Brodal P, Bjaalie JG (1997) Salient anatomic features of the cortico-ponto-cerebellar pathway. Prog Brain Res 114:227–250. 10.1016/S0079-6123(08)63367-1 9193147

[B13] Cahalan M, Neher E (1992) Patch clamp techniques: an overview. Methods Enzymol 207:3–14. 10.1016/0076-6879(92)07003-7 1382186

[B14] Canepari M, Auger C, Ogden D (2004) Ca2+ ion permeability and single-channel properties of the metabotropic slow EPSC of rat Purkinje neurons. J Neurosci 24:3563–3573. 10.1523/JNEUROSCI.5374-03.2004 15071104 PMC6729750

[B15] Cerminara NL, Lang EJ, Sillitoe R V, Apps R (2015) Redefining the cerebellar cortex as an assembly of non-uniform Purkinje cell microcircuits. Nat Rev Neurosci 16:79–93. 10.1038/nrn3886 25601779 PMC4476393

[B16] Chadderton P, Margrie TW, Häusser M (2004) Integration of quanta in cerebellar granule cells during sensory processing. Nature 428:856–860. 10.1038/nature02442 15103377

[B17] Chae HG, Ahn SJ, Hong YH, Chang WS, Kim J, Kim SJ (2012) Transient receptor potential canonical channels regulate the induction of cerebellar long-term depression. J Neurosci 32:12909–12914. 10.1523/JNEUROSCI.0073-12.2012 22973014 PMC6703793

[B18] Cole BA, Becker EBE (2023) Modulation and regulation of canonical transient receptor potential 3 (TRPC3) channels. Cells 12:2215. 10.3390/cells12182215 37759438 PMC10526463

[B19] Crupi R, Impellizzeri D, Cuzzocrea S (2019) Role of metabotropic glutamate receptors in neurological disorders. Front Mol Neurosci 12:1–11. 10.3389/fnmol.2019.00020 30800054 PMC6375857

[B20] De Zeeuw CI (2021) Bidirectional learning in upbound and downbound microzones of the cerebellum. Nat Rev Neurosci 22:92–110. 10.1038/s41583-020-00392-x 33203932

[B21] De Zeeuw CI, Lisberger SG, Raymond JL (2021) Diversity and dynamism in the cerebellum. Nat Neurosci 24:160–167. 10.1038/s41593-020-00754-9 33288911

[B22] Dulneva A, Lee S, Oliver PL, Di Gleria K, Kessler BM, Davies KE, Becker EBE (2015) The mutant moonwalker TRPC3 channel links calcium signaling to lipid metabolism in the developing cerebellum. Hum Mol Genet 24:4114–4125. 10.1093/hmg/ddv150 25908616 PMC4476454

[B23] Eccles JC, Ito M, Szentagothai J (1967) The cerebellum as a neuronal machine. New York: Springer-Verlag.

[B24] Fujita H, Aoki H, Ajioka I, Yamazaki M, Abe M, Oh-Nishi A, Sakimura K, Sugihara I (2014) Detailed expression pattern of aldolase C (aldoc) in the cerebellum, retina and other areas of the CNS studied in Aldoc-Venus knock-in mice. PLoS One 9:e86679. 10.1371/journal.pone.0086679 24475166 PMC3903578

[B25] Fujita H, Morita N, Furuichi T, Sugihara I (2012) Clustered fine compartmentalization of the mouse embryonic cerebellar cortex and its rearrangement into the postnatal striped configuration. J Neurosci 32:15688–15703. 10.1523/JNEUROSCI.1710-12.2012 23136409 PMC6621621

[B26] Furutama D, et al. (2010) Expression of the IP3R1 promoter-driven nls-LacZ transgene in Purkinje cell parasagittal arrays of developing mouse cerebellum. J Neurosci Res 88:2810–2825. 10.1002/jnr.22451 20632399

[B27] Graham DJ, Wylie DR (2012) Zebrin-immunopositive and -immunonegative stripe pairs represent functional units in the pigeon vestibulocerebellum. J Neurosci 32:12769–12779. 10.1523/JNEUROSCI.0197-12.2012 22973000 PMC6703799

[B28] Grandes P, Mateos JM, Ruegg D, Kuhn R, Knopfel T (1994) Differential cellular localization of three splice variants of the mGluRl metabotropic glutamate receptor in rat cerebellum. Neuroreport 5:2249–2252. 10.1097/00001756-199411000-00011 7881038

[B29] Guo C, Huson V, Macosko EZ, Regehr WG (2021) Graded heterogeneity of metabotropic signaling underlies a continuum of cell-intrinsic temporal responses in unipolar brush cells. Nat Commun 12:2–13. 10.1038/s41467-020-20340-8 34620856 PMC8497507

[B30] Hartmann J, et al. (2008) TRPC3 channels are required for synaptic transmission and motor coordination. Neuron 59:392–398. 10.1016/j.neuron.2008.06.009 18701065 PMC2643468

[B31] Hartmann J, Blum R, Kovalchuk Y, Adelsberger H, Kuner R, Durand GM, Miyata M, Kano M, Offermanns S, Konnerth A (2004) Distinct roles of Gαq Gα11 and Purkinje cell signaling and motor behavior. J Neurosci 24:5119–5130. 10.1523/JNEUROSCI.4193-03.2004 15175381 PMC6729195

[B32] Hartmann J, Henning HA, Konnerth A (2011) mGluR1/TRPC3-mediated synaptic transmission and calcium signaling in mammalian central neurons. Cold Spring Harb Perspect Biol 3:1–16. 10.1101/cshperspect.a006726 21441586 PMC3062210

[B33] Hartmann J, Konnerth A (2015) TRPC3-dependent synaptic transmission in central mammalian neurons. J Mol Med 93:983–989. 10.1007/s00109-015-1298-7 26041382

[B34] Hashimoto K, Miyata M, Watanabe M, Kano M (2001) Roles of phospholipase Cβ4 in synapse elimination and plasticity in developing and mature cerebellum. Mol Neurobiol 23:69–82. 10.1385/MN:23:1:69 11642544

[B35] Hirai H, Kano M (2018) Type 1 metabotropic glutamate receptor and its signaling molecules as therapeutic targets for the treatment of cerebellar disorders. Curr Opin Pharmacol 38:51–58. 10.1016/j.coph.2018.02.002 29525719

[B36] Ichise T, Kano M, Hashimoto K, Yanagihara D, Nakao K, Shigemoto R, Katsuki M, Aiba A (2000) Mglur1 in cerebellar Purkinje cells essential for long-term depression, synapse elimination, and motor coordination. Science 288:1832–1835. 10.1126/science.288.5472.1832 10846166

[B37] Ito M (2001) Cerebellar long-term depression: characterization, signal transduction, and functional roles. Physiol Rev 81:1143–1195. 10.1152/physrev.2001.81.3.1143 11427694

[B38] Jorntell H, Ekerot C-F (2006) Properties of somatosensory synaptic integration in cerebellar granule cells in vivo. J Neurosci 26:11786–11797. 10.1523/JNEUROSCI.2939-06.2006 17093099 PMC6674774

[B39] Kano M, Watanabe T (2017) Type-1 metabotropic glutamate receptor signaling in cerebellar Purkinje cells in health and disease. F1000Res 6:1–13. 10.12688/f1000research.10485.1 28435670 PMC5381626

[B40] Kato AS, Knierman MD, Siuda ER, Isaac JTR, Nisenbaum ES, Bredt DS (2012) Glutamate receptor 2 associates with metabotropic glutamate receptor 1 (mGluR1), protein kinase C, and canonical transient receptor potential 3 and regulates mGluR1-mediated synaptic transmission in cerebellar Purkinje neurons. J Neurosci 32:15296–15308. 10.1523/JNEUROSCI.0705-12.2012 23115168 PMC6621574

[B41] Kim SJ (2013) TRPC3 channel underlies cerebellar long-term depression. Cerebellum 12:334–337. 10.1007/s12311-013-0455-1 23408143

[B42] Kim CH, Oh SH, Lee JH, Chang SO, Kim J, Kim SJ (2012a) Lobule-specific membrane excitability of cerebellar Purkinje cells. J Physiol 590:273–288. 10.1113/jphysiol.2011.221846 22083600 PMC3285064

[B43] Kim Y, Wong ACY, Power JM, Tadros SF, Klugmann M, Moorhouse AJ, Bertrand PP, Housley GD (2012b) Alternative splicing of the TRPC3 ion channel calmodulin/ IP 3 receptor-binding domain in the hindbrain enhances cation flux. J Neurosci 32:11414–11423. 10.1523/JNEUROSCI.6446-11.2012 22895723 PMC6621195

[B44] Kishimoto Y, Fujimichi R, Araishi K, Kawahara S, Kano M, Aiba A, Kirino Y (2002) mGluR1 in cerebellar Purkinje cells is required for normal association of temporally contiguous stimuli in classical conditioning. Eur J Neurosci 16:2416–2424. 10.1046/j.1460-9568.2002.02407.x 12492436

[B45] Knöpfel T, Grandes P (2002) Metabotropic glutamate receptors in the cerebellum with a focus on their function in Purkinje cells. Cerebellum 1:19–26. 10.1007/BF02941886 12879970

[B46] Kozareva V, Martin C, Osorno T, Rudolph S, Guo C, Vanderburg C, Nadaf N, Regev A, Regehr WG, Macosko E (2021) A transcriptomic atlas of mouse cerebellar cortex comprehensively defines cell types. Nature 598:214–219. 10.1038/s41586-021-03220-z 34616064 PMC8494635

[B47] Livak KJ, Schmittgen TD (2001) Analysis of relative gene expression data using real-time quantitative PCR and the 2-ΔΔCT method. Methods 25:402–408. 10.1006/meth.2001.1262 11846609

[B48] Lüscher C, Huber KM (2010) Group 1 mGluR-dependent synaptic long-term depression: mechanisms and implications for circuitry and disease. Neuron 65:445–459. 10.1016/j.neuron.2010.01.016 20188650 PMC2841961

[B49] Mateos JM, et al. (2001) Parasagittal compartmentalization of the metabotropic glutamate receptor mGluR1b in the cerebellar cortex. Eur J Anat 5:15–21.

[B50] Meera P, Pulst S, Otis T (2017) A positive feedback loop linking enhanced mGluR function and basal calcium in spinocerebellar ataxia type 2. Elife 6:1–14. 10.7554/eLife.26377 28518055 PMC5444899

[B51] Mercer AA, Palarz KJ, Tabatadze N, Woolley CS, Raman IM (2016) Sex differences in cerebellar synaptic transmission and sex-specific responses to autism-linked Gabrb3 mutations in mice. 10.7554/eLife.07596 PMC4878876 27077953

[B52] Miyata M, et al. (2001) Deficient long-term synaptic depression in the rostral cerebellum correlated with impaired motor learning in phospholipase C β4 mutant mice. Eur J Neurosci 13:1945–1954. 10.1046/j.0953-816x.2001.01570.x 11403688

[B53] Nelson C, Glitsch MD (2012) Lack of kinase regulation of canonical transient receptor potential 3 (TRPC3) channel-dependent currents in cerebellar Purkinje cells. J Biol Chem 287:6326–6335. 10.1074/jbc.M111.246553 22207762 PMC3307326

[B54] Nguyen-Minh VT, Tran-Anh K, Luo Y, Sugihara I (2019) Electrophysiological excitability and parallel fiber synaptic properties of zebrin-positive and -negative Purkinje cells in lobule VIII of the mouse cerebellar slice. Front Cell Neurosci 12:1–11. 10.3389/fncel.2018.00513 30670950 PMC6331690

[B55] Ohtani Y, et al. (2014) The synaptic targeting of mGluR1 by its carboxyl-terminal domain is crucial for cerebellar function. J Neurosci 34:2702–2712. 10.1523/JNEUROSCI.3542-13.2014 24523559 PMC6802745

[B56] Ouares KA, Canepari M (2020) The origin of physiological local mGluR1 supralinear Ca2+ signals in cerebellar Purkinje neurons. J Neurosci 40:1795–1809. 10.1523/JNEUROSCI.2406-19.2020 31969470 PMC7046445

[B57] Perkins KL (2006) Cell-attached voltage-clamp and current-clamp recording and stimulation techniques in brain slices. J Neurosci Methods 154:1–18. 10.1016/j.jneumeth.2006.02.010 16554092 PMC2373773

[B58] Person AL, Raman IM (2012) Purkinje neuron synchrony elicits time-locked spiking in the cerebellar nuclei. Nature 481:502–505. 10.1038/nature10732 22198670 PMC3268051

[B59] Pijpers A, Apps R, Pardoe J, Voogd J, Ruigrok TJH (2006) Precise spatial relationships between mossy fibers and climbing fibers in rat cerebellar cortical zones. J Neurosci 26:12067–12080. 10.1523/JNEUROSCI.2905-06.2006 17108180 PMC6674858

[B60] Proville RD, Spolidoro M, Guyon N, Dugué GP, Selimi F, Isope P, Popa D, Léna C (2014) Cerebellum involvement in cortical sensorimotor circuits for the control of voluntary movements. Nat Neurosci 17:1233–1239. 10.1038/nn.3773 25064850

[B61] Rae JL, Fernandez J (1988) Perforated patch recordings in physiology. Physiology 6:273–276.

[B62] Rodriques SG, Stickels RR, Goeva A, Martin CA, Murray E, Vanderburg CR, Welch J, Chen LM, Chen F, Macosko EZ (2019) Slide-seq: a scalable technology for measuring genome-wide expression at high spatial resolution. Science 363:1463–1467. 10.1126/science.aaw1219 30923225 PMC6927209

[B63] Ruigrok TJH (2011) Ins and outs of cerebellar modules. Cerebellum 10:464–474. 10.1007/s12311-010-0164-y 20232190 PMC3169761

[B64] Sakmann B, Neher E (1984) Patch clamp techniques for studying ionic channels in excitable membranes. Annu Rev Physiol 46:455–472. 10.1146/annurev.ph.46.030184.002323 6143532

[B65] Sarna JR, Marzban H, Watanabe M, Hawkes R (2006) Complementary stripes of phospholipase Cβ3 and Cβ4 expression by Purkinje cell subsets in the mouse cerebellum. J Comp Neurol 496:303–313. 10.1002/cne.20912 16566000

[B66] Schmahmann JD (2010) The role of the cerebellum in cognition and emotion: personal reflections since 1982 on the dysmetria of thought hypothesis, and its historical evolution from theory to therapy. Neuropsychol Rev 20:236–260. 10.1007/s11065-010-9142-x 20821056

[B67] Sedaghat-Nejad E, Pi JS, Hage P, Fakharian MA, Shadmehr R (2022) Synchronous spiking of cerebellar Purkinje cells during control of movements. Proc Natl Acad Sci U S A 119:e2118954119. 10.1073/pnas.2118954119 35349338 PMC9168948

[B68] Sekerková G, Kim JA, Nigro MJ, Becker EBE, Hartmann J, Birnbaumer L, Mugnaini E, Martina M (2013) Early onset of ataxia in moonwalker mice is accompanied by complete ablation of type II unipolar brush cells and Purkinje cell dysfunction. J Neurosci 33:19689–19694. 10.1523/JNEUROSCI.2294-13.2013 24336732 PMC3858636

[B69] Shimuta M, Sugihara I, Ishikawa T (2020) Multiple signals evoked by unisensory stimulation converge onto cerebellar granule and Purkinje cells in mice. Commun Biol 3:1–12. 10.1038/s42003-020-1110-2 32669638 PMC7363865

[B70] Sierra-Valdez F, Azumaya CM, Romero LO, Nakagawa T, Cordero-Morales JF (2018) Structure–function analyses of the ion channel TRPC3 reveal that its cytoplasmic domain allosterically modulates channel gating. J Biol Chem 293:16102–16114. 10.1074/jbc.RA118.005066 30139744 PMC6187627

[B71] Song M, Chen D, Yu SP (2014) The TRPC channel blocker SKF 96365 inhibits glioblastoma cell growth by enhancing reverse mode of the Na+/Ca2+ exchanger and increasing intracellular Ca2+. Br J Pharmacol 171:3432–3447. 10.1111/bph.12691 24641279 PMC4105931

[B72] Steele CJ, Chakravarty MM (2018) Gray-matter structural variability in the human cerebellum: lobule-specific differences across sex and hemisphere. Neuroimage 170:164–173. 10.1016/j.neuroimage.2017.04.066 28461060

[B73] Stoodley CJ, Valera EM, Schmahmann JD (2012) Functional topography of the cerebellum for motor and cognitive tasks: an fMRI study. Neuroimage 59:1560–1570. 10.1016/j.neuroimage.2011.08.065 21907811 PMC3230671

[B74] Sugihara I, Shinoda Y (2004) Molecular, topographic, and functional organization of the cerebellar cortex: a study with combined aldolase C and olivocerebellar labeling. J Neurosci 24:8771–8785. 10.1523/JNEUROSCI.1961-04.2004 15470143 PMC6729951

[B75] Suvrathan A, Payne HL, Raymond JL (2016) Timing rules for synaptic plasticity matched to behavioral function. Neuron 92:959–967. 10.1016/j.neuron.2016.10.022 27839999 PMC5165237

[B76] Suvrathan A, Raymond JL (2018) Depressed by learning—heterogeneity of the plasticity rules at parallel fiber synapses onto Purkinje cells. Cerebellum 17:747–755. 10.1007/s12311-018-0968-8 30069835 PMC6550343

[B77] Takechi H, Eilers J, Konnerth A (1998) A new class of synaptic response involving calcium release in dendritic spines. Nature 396:757–760. 10.1038/25547 9874373

[B78] Telgkamp P, Raman IM (2002) Depression of inhibitory synaptic transmission between Purkinje cells and neurons of the cerebellar nuclei. J Neurosci 22:8447–8457. 10.1523/JNEUROSCI.22-19-08447.2002 12351719 PMC6757792

[B79] Tempia F, Miniaci MC, Anchisi D, Strata P (1998) Postsynaptic current mediated by metabotropic glutamate receptors in cerebellar Purkinje cells. J Neurophysiol 80:520–528. 10.1152/jn.1998.80.2.520 9705447

[B80] Tian J, Zhu M (2018) GABAB receptors augment TRPC3-mediated slow excitatory postsynaptic current to regulate cerebellar Purkinje neuron response to type-1 metabotropic glutamate receptor activation. Cells 7:90. 10.3390/cells7080090 30060610 PMC6116156

[B81] Tiapko O, Groschner K (2018) TRPC3 as a target of novel therapeutic interventions. Cells 7:83. 10.3390/cells7070083 30037143 PMC6071100

[B82] Tsutsumi S, Yamazaki M, Miyazaki T, Watanabe M, Sakimura K, Kano M, Kitamura K (2015) Structure-function relationships between aldolase C/zebrin II expression and complex spike synchrony in the cerebellum. J Neurosci 35:843–852. 10.1523/JNEUROSCI.2170-14.2015 25589776 PMC6605375

[B83] Valera AM, Binda F, Pawlowski SA, Dupont JL, Casella JF, Rothstein JD, Poulain B, Isope P (2016) Stereotyped spatial patterns of functional synaptic connectivity in the cerebellar cortex. Elife 5:1–22. 10.7554/eLife.09862 26982219 PMC4805550

[B84] van Beugen BJ, Gao Z, Boele H-J, Hoebeek F, De Zeeuw CI (2013) High frequency burst firing of granule cells ensures transmission at the parallel fiber to Purkinje cell synapses at the cost of temporal coding. Front Neural Circuits 7:95. 10.3389/fncir.2013.00095 23734102 PMC3659283

[B85] Vazquez G, Wedel BJ, Kawasaki BT, St. John Bird G, Putney JW (2004) Obligatory role of Src kinase in the signaling mechanism for TRPC3 cation channels. J Biol Chem 279:40521–40528. 10.1074/jbc.M405280200 15271991

[B86] Voogd J (2011) Cerebellar zones: a personal history. Cerebellum 10:334–350. 10.1007/s12311-010-0221-6 20967577 PMC3169774

[B87] Voogd J, Ruigrok TJH (2004) The organization of the corticonuclear and olivocerebellar climbing fiber projections to the rat cerebellar vermis: the congruence of projection zones and the zebrin pattern. J Neurocytol 33:5–21. 10.1023/B:NEUR.0000029645.72074.2b 15173629

[B88] Wadiche JI, Jahr CE (2005) Patterned expression of Purkinje cell glutamate transporters controls synaptic plasticity. Nat Neurosci 8:1329–1334. 10.1038/nn1539 16136036

[B89] Wagner MJ, Hyun Kim T, Savall J, Schnitzer MJ, Luo L (2017) Cerebellar granule cells encode the expectation of reward. Nature 544:96–100. 10.1038/nature21726 28321129 PMC5532014

[B90] Wang SS, Denk W, Häusser M (2000) Coincidence detection in single dendritic spines mediated by calcium release. Nat Neurosci 3:1266–1273. 10.1038/81792 11100147

[B91] Wiestler T, McGonigle DJ, Diedrichsen J (2011) Integration of sensory and motor representations of single fingers in the human cerebellum. J Neurophysiol 105:3042–3053. 10.1152/jn.00106.2011 21471398

[B92] Witter L, De Zeeuw CI (2015a) Regional functionality of the cerebellum. Curr Opin Neurobiol 33:150–155. 10.1016/j.conb.2015.03.017 25884963

[B93] Witter L, De Zeeuw CI (2015b) In vivo differences in inputs and spiking between neurons in lobules VI/VII of neocerebellum and lobule X of archaeocerebellum. The Cerebellum 14:506–515. 10.1007/s12311-015-0654-z 25735968 PMC4612334

[B94] Wu B, et al. (2019) TRPC3 is a major contributor to functional heterogeneity of cerebellar Purkinje cells. Elife 8:1–31. 10.7554/eLife.45590 31486767 PMC6733575

[B95] Yamakawa Y, Hirano T (1999) Contribution of mGluR1 to the basal activity of a mouse cerebellar Purkinje neuron. Neurosci Lett 277:103–106. 10.1016/S0304-3940(99)00852-6 10624820

[B96] Yamasaki M, Aiba A, Kano M, Watanabe M (2021) mGluR1 signaling in cerebellar Purkinje cells: subcellular organization and involvement in cerebellar function and disease. Neuropharmacology 194:108629. 10.1016/j.neuropharm.2021.108629 34089728

[B97] Yu Z, Guindani M, Grieco SF, Chen L, Holmes TC, Xu X (2022) Beyond *t* test and ANOVA: applications of mixed-effects models for more rigorous statistical analysis in neuroscience research. Neuron 110:21–35. 10.1016/j.neuron.2021.10.030 34784504 PMC8763600

[B98] Zhou H, Lin Z, Voges K, Ju C, Gao Z, Bosman LWJ, Ruigrok TJ, Hoebeek FE, De Zeeuw CI, Schonewille M (2014) Cerebellar modules operate at different frequencies. Elife 2014:1–18. 10.7554/eLife.02536 24843004 PMC4049173

[B99] Zhou H, Voges K, Lin Z, Ju C, Schonewille M (2015) Differential Purkinje cell simple spike activity and pausing behavior related to cerebellar modules. J Neurophysiol 113:2524–2536. 10.1152/jn.00925.2014 25717166 PMC4416590

