# Supplementary figures and images for: Heterogeneity in Slow Synaptic Transmission Diversifies Purkinje Cell Timing
Source: J Neurosci. 2024 Aug 15;44(37):e0455242024. doi: 10.1523/JNEUROSCI.0455-24.2024 (PMC11391503; doi:10.1523/JNEUROSCI.0455-24.2024)

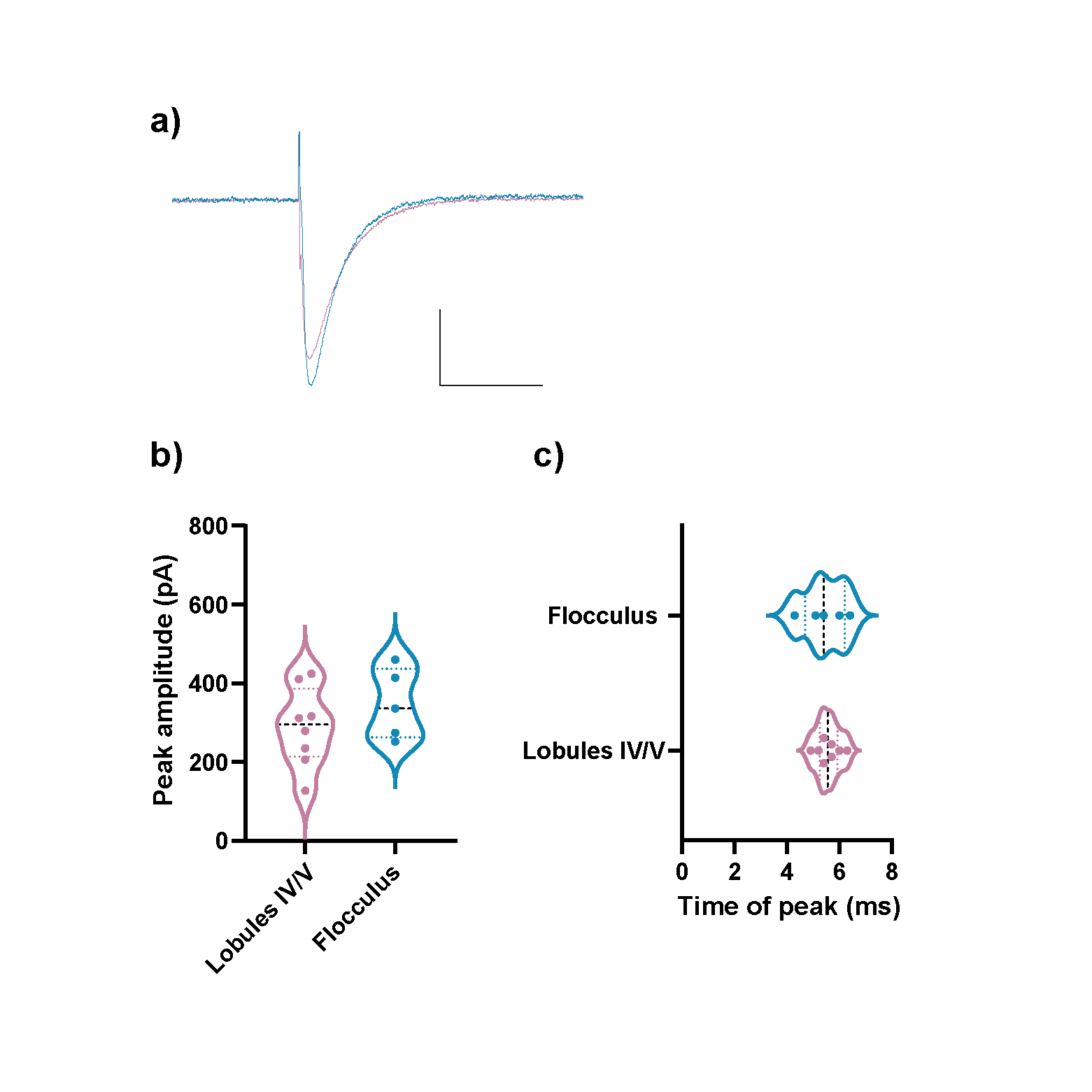

Supplement: Figure 1-1 — AMPAR-mediated synaptic responses in lobules IV/V and flocculus were indistinguishable. a) Representative traces of AMPAR-mediated EPSC from lobules IV/V and the flocculus. Scale bar 100 pA, 50 ms. Peak amplitude (b) and time of peak (c) were the same in lobules IV/V and the flocculus, in contrast to the time of peak of sEPSCs. Violin plots show median and quartiles. (Fig 1-1 b,c: Lobules IV/V = 8 cells from 4 mice, flocculus = 5 cells from 5 mice.) Download Figure 1-1, TIF file. [file jneuro-44-e0455242024-s001.tif]

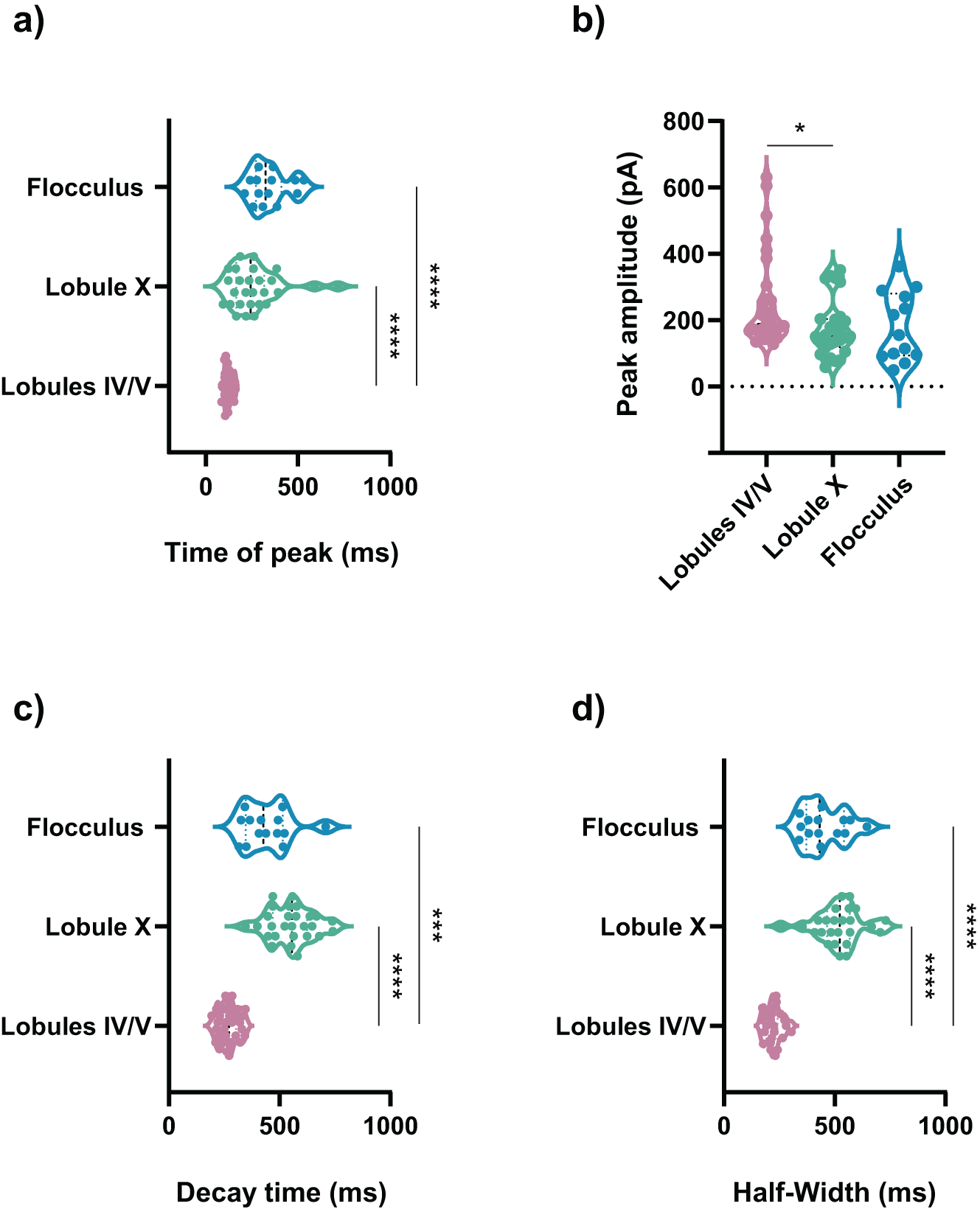

Supplement: Figure 1-2 — Lobule-specific synaptic dynamics were not sensitive to Purkinje cell dialysis. a) Time of peak of a subset of sEPSCs from Figure 1, across lobules IV/V, lobule X, and the flocculus, recorded within 10-20 min after break-in to whole-cell configuration, demonstrated shorter time of peak in lobules IV/V. b) Peak amplitude of sEPSCs was similar across regions, even for the subset of cells. c) Decay time of sEPSCs was shorter in lobules IV/V, in comparison to lobule X and the flocculus, even for the subset of cells. d) Half-widths of sEPSCs were shorter in lobules IV/V, in comparison to lobule X and the flocculus, even for the subset of cells. Statistical comparisons: (a,b,c,d)*p < 0.05, ***p < 0.001, ****p < 0.0001, Kruskal-Wallis test followed by Dunn’s multiple comparisons test. Violin plots show median and quartiles. (Fig 1-2 a-c: lobules IV/V = 31 cells from 27 mice, lobule X = 27 cells from 22 mice; flocculus = 14 cells from 11 mice. Fig 1-2 d: lobules IV/V = 23 cells from 21 mice, lobule X = 25 cells from 21 mice; flocculus = 14 cells from 11 mice.) Download Figure 1-2, TIF file. [file jneuro-44-e0455242024-s002.tif]

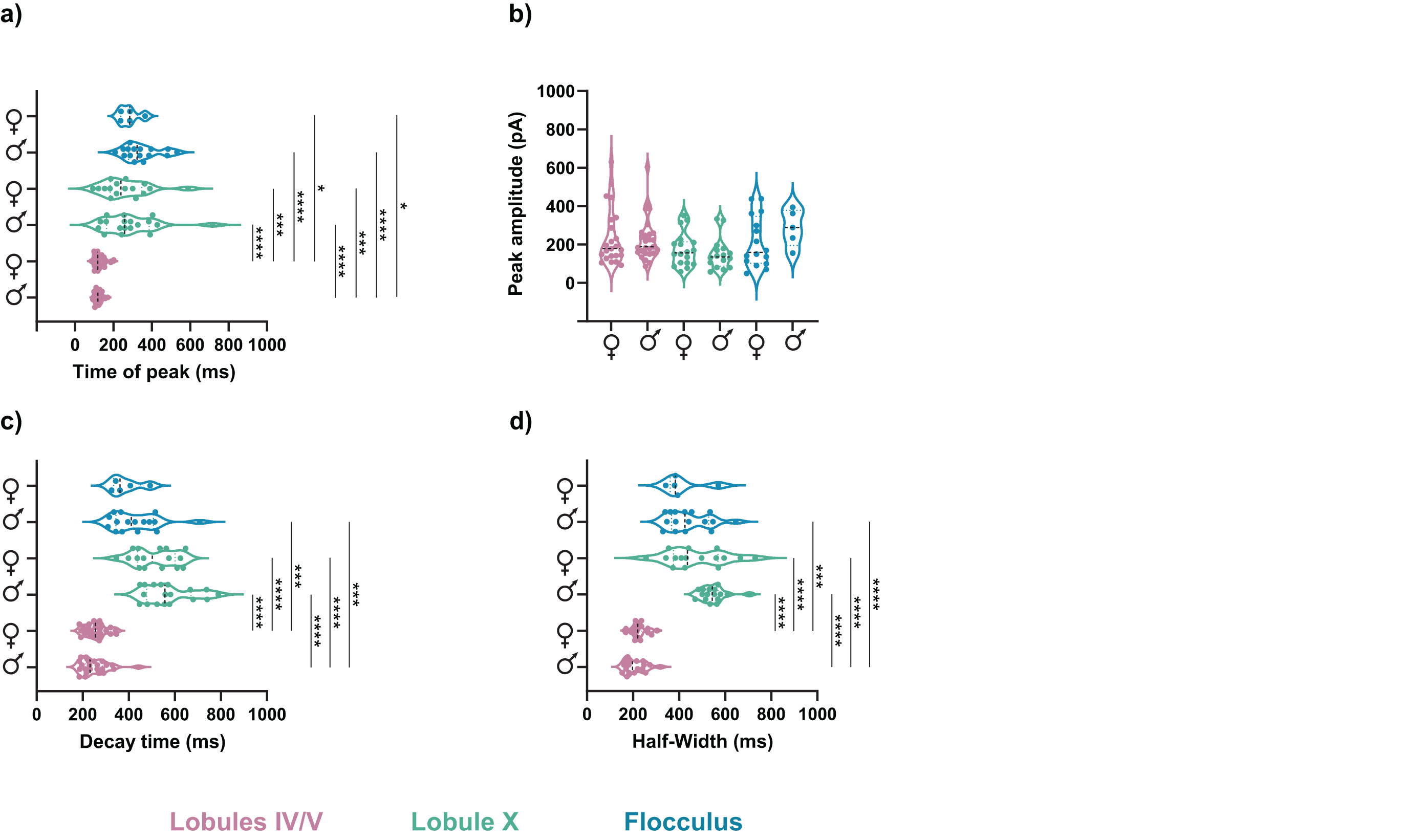

Supplement: Figure 1-3 — Animal sex did not determine lobule-specific synaptic dynamics. a) Subset analysis of data in Figure 1 demonstrated that the sEPSC time of peak did not differ across sexes, nor did peak amplitude (b), decay time (c), or half-width (d). Statistical comparisons: (a,b,c,d) *p < 0.05, ***p < 0.001, ****p < 0.0001, Kruskal-Wallis test followed by Dunn’s multiple comparisons test. (Fig 1-3 a-c: Lobules IV/V male = 23 cells from 17 mice, Lobules IV/V female = 27 cells from 20 mice, lobule X male = 18 cells from 13 mice; lobule X female = 16 cells from 13 mice, flocculus male = 16 cells from 8 mice, flocculus female = 5 cells from 4 mice; Fig 1-3 d: Lobules IV/V male = 18 cells from 13 mice, Lobules IV/V female = 19 cells from 16 mice, lobule X male = 16 cells from 12 mice; lobule X female = 16 cells from 13 mice, flocculus male = 16 cells from 8 mice, flocculus female = 5 cells from 4 mice.) Download Figure 1-3, TIF file. [file jneuro-44-e0455242024-s003.tif]

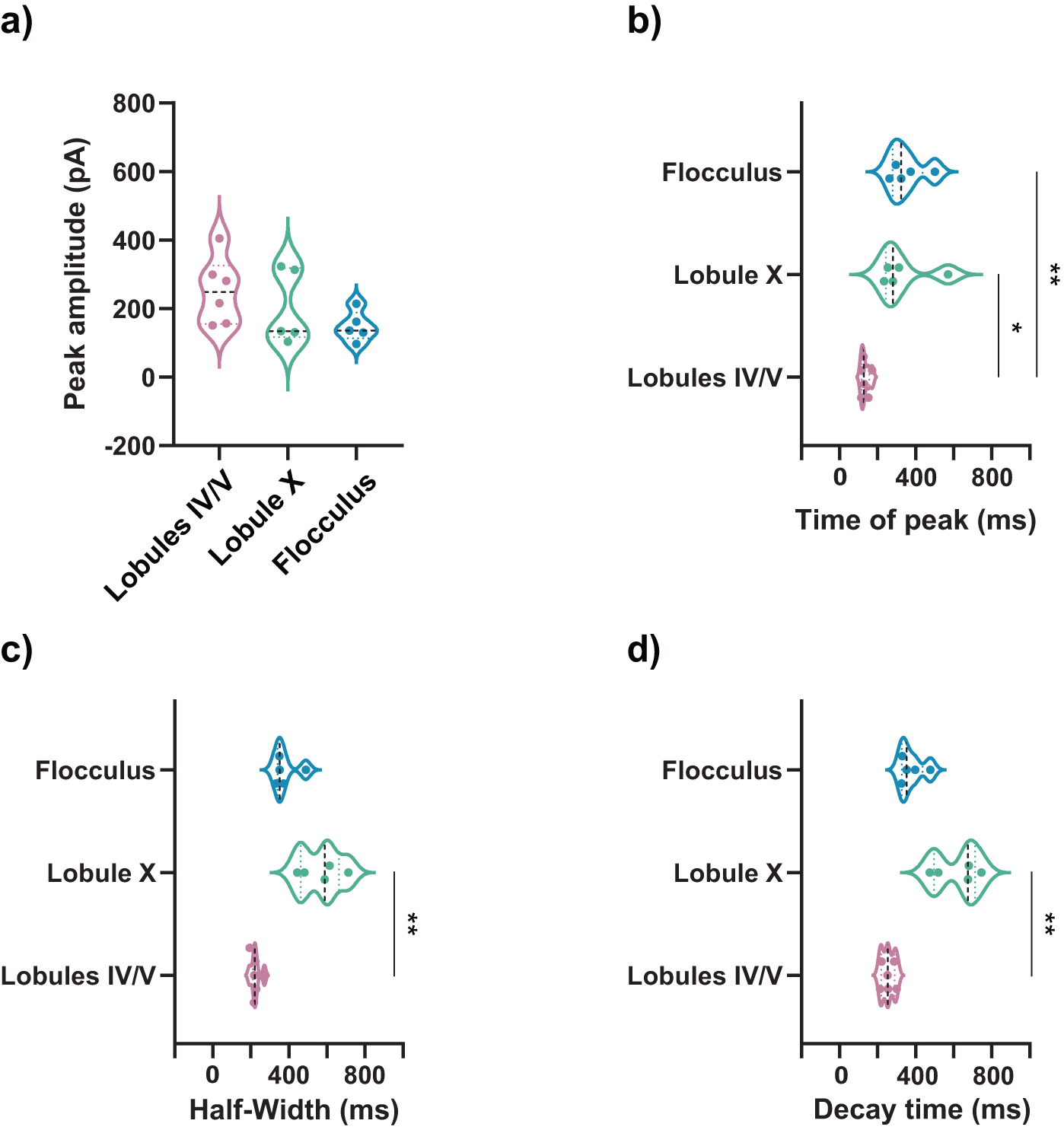

Supplement: Figure 6-1 — Buffering intracellular calcium did not change heterogeneity in synaptic timing. sEPSC recordings were performed with 10 mM EGTA and 1mM BAPTA added to the internal solution. a) Peak amplitude of the sEPSC did not vary between lobules (b-d) However, heterogeneity in the time of peak, half-width, and decay time of the sEPSC persisted. Statistical comparisons: (a, b, c, d) *p < 0.05, **p < 0.01, Kruskal-Wallis test followed by Dunn’s multiple comparisons test. (Fig 6-1 a-d: Lobules IV/V = 6 cells from 4 mice, lobule X = 5 cells from 3 mice, flocculus = 5 cells from 3 mice) Download Figure 6-1, TIF file. [file jneuro-44-e0455242024-s004.tif]
