# Supplementary material for: Heterogeneity in Slow Synaptic Transmission Diversifies Purkinje Cell Timing
Source: J Neurosci. 2024 Aug 15;44(37):e0455242024. doi: 10.1523/JNEUROSCI.0455-24.2024 (PMC11391503; doi:10.1523/JNEUROSCI.0455-24.2024)
Supplement: Table 1 — Details on statistical analyses. Download Table 1, DOCX file. [file jneuro-44-e0455242024-s005.docx]

**Extended Data Table 1.** Details on statistical analyses

| **Figure** | **Statistical analysis** | **Statistic** | **p-Value** | **Effect size** | **Post-hoc pairwise comparison (only significant effects described)** | **p-Value** |
| --- | --- | --- | --- | --- | --- | --- |
| 1 c | Kruskal-Wallis test | H (2) = 8.371 | 0.0152 | 0.057 | Dunn’s multiple comparisons test |  |
|  |  |  |  |  | Lobule IV/V vs. Lobule X | 0.0120 |
| 1 d | Kruskal-Wallis test | H (2) = 72.71 | <0.0001 | 0.637 | Dunn’s multiple comparisons test |  |
|  |  |  |  |  | Lobule IV/V vs. Lobule X | <0.0001 |
|  |  |  |  |  | Lobule IV/V vs. Flocculus | <0.0001 |
| 1 f | Kruskal-Wallis test | H (2) = 85.87 | <0.0001 | 0.756 | Dunn’s multiple comparisons test |  |
|  |  |  |  |  | Lobule IV/V vs. Lobule X | <0.0001 |
|  |  |  |  |  | Lobule IV/V vs. Flocculus | <0.0001 |
|  |  |  |  |  | Lobule X vs. Flocculus | 0.0463 |
| 1 g | Kruskal-Wallis test | H (2) = 73.34 | <0.0001 | 0.751 | Dunn’s multiple comparisons test |  |
|  |  |  |  |  | Lobule IV/V vs. Lobule X | <0.0001 |
|  |  |  |  |  | Lobule IV/V vs. Flocculus | <0.0001 |
| 3 a | Kruskal-Wallis test | H (2) =2.676 | 0.2801 |  |  |  |
| 3 b | Ordinary one-way ANOVA | F (2,19) = 3.310 | 0.0584 |  |  |  |
| 3 c | Kruskal-Wallis test | H (2) =1.244 | 0.5369 |  |  |  |
| 3 d | Kruskal-Wallis test | H (2) =9.693 | 0.0017 | 0.592 | Dunn’s multiple comparisons test |  |
|  |  |  |  |  | Lobule IV/V vs. Flocculus | 0.0057 |
| 3 e | Ordinary one-way ANOVA | F (2,19) = 17.88 | <0.0001 | 0.605 | Tukey’s multiple comparisons test |  |
|  |  |  |  |  | Lobule IV/V vs. Lobule X | 0.0144 |
|  |  |  |  |  | Lobule IV/V vs. Flocculus | <0.0001 |
|  |  |  |  |  | Lobule X vs. Flocculus | 0.0252 |
| 3 f | Ordinary one-way ANOVA | F (2, 20) = 23.10 | <0.0001 | 0.658 | Tukey’s multiple comparisons test |  |
|  |  |  |  |  | Lobule IV/V vs. Lobule X | 0.0402 |
|  |  |  |  |  | Lobule IV/V vs. Flocculus | <0.0001 |
|  |  |  |  |  | Lobule X vs. Flocculus | 0.0016 |
| 3 g | Kruskal-Wallis test | H (2) =7.585 | 0.0110 | 0.430 | Dunn’s multiple comparisons test |  |
|  |  |  |  |  | Lobule IV/V vs. Lobule X | 0.0177 |
| 3 h | Ordinary one-way ANOVA | F (2, 19) =16.45 | <0.0001 | 0.584 | Tukey’s multiple comparisons test |  |
|  |  |  |  |  | Lobule IV/V vs. Lobule X | <0.0001 |
|  |  |  |  |  | Lobule X vs. Flocculus | 0.0027 |
| 3 i | Kruskal-Wallis test | H (2) = 16.63 | 0.0002 | 0.732 | Dunn’s multiple comparisons test |  |
|  |  |  |  |  | Lobule IV/V vs. Lobule X | 0.0002 |
|  |  |  |  |  | Lobule X vs. Flocculus | 0.0269 |
| 4 a (50 Hz) | Kruskal-Wallis test | H (2)= 2.002 | 0.3675 |  |  |  |
| 4 a (75 Hz) | Kruskal-Wallis test | H (2)= 1.048 | 0.5923 |  |  |  |
| 4 a (100 Hz) | Kruskal-Wallis test | H (2)= 2.865 | 0.2387 |  |  |  |
| 4 a (125 Hz) | Kruskal-Wallis test | H (2)= 3.848 | 0.1460 |  |  |  |
| 4 a (150 Hz) | Kruskal-Wallis test | H (2)= 5.220 | 0.0735 |  |  |  |
| 4 a (200Hz) | Kruskal-Wallis test | H (2)= 11.92 | 0.0026 | 0.220 | Dunn’s multiple comparisons test |  |
|  |  |  |  |  | Lobule IV/V vs. Lobule X | 0.0021 |
| 4 b (50 Hz) | Kruskal-Wallis test | H (2)= 36.03 | <0.0001 | 0.740 | Dunn’s multiple comparisons test |  |
|  |  |  |  |  | Lobule IV/V vs. Lobule X | <0.0001 |
|  |  |  |  |  | Lobule IV/V vs. Flocculus | <0.0001 |
| 4 b (75 Hz) | Kruskal-Wallis test | H (2)= 37.17 | <0.0001 | 0.733 | Dunn’s multiple comparisons test |  |
|  |  |  |  |  | Lobule IV/V vs. Lobule X | <0.0001 |
|  |  |  |  |  | Lobule IV/V vs. Flocculus | <0.0001 |
| 4 b (100 Hz) | Kruskal-Wallis test | H (2)= 38.95 | <0.0001 | 0.739 | Dunn’s multiple comparisons test |  |
|  |  |  |  |  | Lobule IV/V vs. Lobule X | <0.0001 |
|  |  |  |  |  | Lobule IV/V vs. Flocculus | <0.0001 |
| 4 b (125 Hz) | Kruskal-Wallis test | H (2)= 36.76 | <0.0001 | 0.740 | Dunn’s multiple comparisons test |  |
|  |  |  |  |  | Lobule IV/V vs. Lobule X | <0.0001 |
|  |  |  |  |  | Lobule IV/V vs. Flocculus | <0.0001 |
| 4 b (150 Hz) | Kruskal-Wallis test | H (2)= 36.91 | <0.0001 | 0.743 | Dunn’s multiple comparisons test |  |
|  |  |  |  |  | Lobule IV/V vs. Lobule X | <0.0001 |
|  |  |  |  |  | Lobule IV/V vs. Flocculus | <0.0001 |
| 4 b (200 Hz) | Kruskal-Wallis test | H (2)= 35.34 | <0.0001 | 0.741 | Dunn’s multiple comparisons test |  |
|  |  |  |  |  | Lobule IV/V vs. Lobule X | <0.0001 |
|  |  |  |  |  | Lobule IV/V vs. Flocculus | <0.0001 |
| 4 c (50 Hz) | Kruskal-Wallis test | H (2)= 38.08 | <0.0001 | 0.784 | Dunn’s multiple comparisons test |  |
|  |  |  |  |  | Lobule IV/V vs. Lobule X | <0.0001 |
|  |  |  |  |  | Lobule IV/V vs. Flocculus | 0.0231 |
| 4 c (75 Hz) | Kruskal-Wallis test | H (2)= 39.65 | <0.0001 | 0.784 | Dunn’s multiple comparisons test |  |
|  |  |  |  |  | Lobule IV/V vs. Lobule X | <0.0001 |
|  |  |  |  |  | Lobule IV/V vs. Flocculus | 0.0148 |
| 4 c (100 Hz) | Ordinary one-way ANOVA | F (2, 50)= 115.7 | <0.0001 | 0.812 | Tukey’s multiple comparisons test |  |
|  |  |  |  |  | Lobule IV/V vs. Lobule X | <0.0001 |
|  |  |  |  |  | Lobule IV/V vs. Flocculus | <0.0001 |
|  |  |  |  |  | Lobule X vs. Flocculus | <0.0001 |
| 4 c (125 Hz) | Ordinary one-way ANOVA | F (2, 47)= 141.5 | <0.0001 | 0.849 | Tukey’s multiple comparisons test |  |
|  |  |  |  |  | Lobule IV/V vs. Lobule X | <0.0001 |
|  |  |  |  |  | Lobule IV/V vs. Flocculus | <0.0001 |
|  |  |  |  |  | Lobule X vs. Flocculus | <0.0001 |
| 4 c (150 Hz) | Ordinary one-way ANOVA | F (2, 47)= 94.50 | <0.0001 | 0.789 | Tukey’s multiple comparisons test |  |
|  |  |  |  |  | Lobule IV/V vs. Lobule X | <0.0001 |
|  |  |  |  |  | Lobule IV/V vs. Flocculus | <0.0001 |
|  |  |  |  |  | Lobule X vs. Flocculus | <0.0001 |
| 4 c (200 Hz) | Ordinary one-way ANOVA | F (2, 45)= 72.39 | <0.0001 | 0.748 | Tukey’s multiple comparisons test |  |
|  |  |  |  |  | Lobule IV/V vs. Lobule X | <0.0001 |
|  |  |  |  |  | Lobule IV/V vs. Flocculus | <0.0001 |
|  |  |  |  |  | Lobule X vs. Flocculus | 0.0002 |
| 5 a | Paired t test | t (11) = 8.366 | <0.0001 | 2.415 |  |  |
| 5 b | Wilcoxon matched-pairs signed rank test | Z=-2.666 | 0.0039 | 0.889 |  |  |
| 5 c | Wilcoxon matched-pairs signed rank test | Z=-2.934 | 0.0010 | 0.885 |  |  |
| 7 a | Ordinary one-way ANOVA | F (2, 39)= 18.08 | <0.0001 | 0.448 | Tukey’s multiple comparisons test |  |
|  |  |  |  |  | Lobule IV/V vs. Lobule X | 0.0325 |
|  |  |  |  |  | Lobule IV/V vs. Flocculus | <0.0001 |
| 7 b | Ordinary one-way ANOVA | F (2, 39)= 16.38 | <0.0001 | 0.423 | Tukey’s multiple comparisons test |  |
|  |  |  |  |  | Lobule IV/V vs. Lobule X | 0.0002 |
|  |  |  |  |  | Lobule X vs. Flocculus | <0.0001 |
| 7 c | Ordinary one-way ANOVA | F (2, 39)= 28.42 | <0.0001 | 0.566 | Tukey’s multiple comparisons test |  |
|  |  |  |  |  | Lobule IV/V vs. Lobule X | <0.0001 |
|  |  |  |  |  | Lobule IV/V vs. Flocculus | <0.0001 |
| 7 d | Kruskal-Wallis test | H (2)= 2.517 | 0.2841 |  |  |  |
| 7 e | Kruskal-Wallis test | H (2)= 0.1808 | 0.9136 |  |  |  |
| 7 f | Kruskal-Wallis test | H (2)= 26.55 | <0.0001 | 0.646 | Dunn’s multiple comparisons test |  |
|  |  |  |  |  | Lobule IV/V vs. Lobule X | 0.0001 |
|  |  |  |  |  | Lobule IV/V vs. Flocculus | <0.0001 |
| 8 c | Ordinary one-way ANOVA | F (2, 21) = 75.71 | <0.0001 | 0.862 | Tukey’s multiple comparisons test |  |
|  |  |  |  |  | Lobule IV/V vs. Lobule X | <0.0001 |
|  |  |  |  |  | Lobule IV/V vs. Flocculus | <0.0001 |
| Extended Data 1-1 b | Unpaired t test | t (11)= 1.064 | 0.3102 |  |  |  |
| Extended Data 1-1 c | Unpaired t test | t (11)= 0.3904 | 0.7037 |  |  |  |
| Extended Data 1-2 a | Kruskal-Wallis test | H (2) = 44.64 | <0.0001 | 0.618 | Dunn’s multiple comparisons test |  |
|  |  |  |  |  | Lobule IV/V vs. Lobule X | <0.0001 |
|  |  |  |  |  | Lobule IV/V vs. Flocculus | <0.0001 |
| Extended Data 1-2 b | Kruskal-Wallis test | H (2) =7.689 | 0.0214 | 0.082 | Dunn’s multiple comparisons test |  |
|  |  |  |  |  | Lobule IV/V vs. Lobule X | 0.0309 |
| Extended Data 1-2 c | Kruskal-Wallis test | H (2) = 53.72 | <0.0001 | 0.750 | Dunn’s multiple comparisons test |  |
|  |  |  |  |  | Lobule IV/V vs. Lobule X | <0.0001 |
|  |  |  |  |  | Lobule IV/V vs. Flocculus | 0.0002 |
| Extended Data 1-2 d | Kruskal-Wallis test | H (2) = 43.52 | <0.0001 | 0.704 | Dunn’s multiple comparisons test |  |
|  |  |  |  |  | Lobule IV/V vs. Lobule X | <0.0001 |
|  |  |  |  |  | Lobule IV/V vs. Flocculus | <0.0001 |
| Extended Data 1-3 a | Kruskal-Wallis test | H (5) = 66.35 | <0.0001 | 0.620 | Dunn’s multiple comparisons test |  |
|  |  |  |  |  | Lobule IV/V male vs. Lobule X male | <0.0001 |
|  |  |  |  |  | Lobule IV/V male vs. Lobule X female | 0.0007 |
|  |  |  |  |  | Lobule IV/V male vs. Flocculus male | <0.0001 |
|  |  |  |  |  | Lobule IV/V male vs. Flocculus female | 0.0174 |
|  |  |  |  |  | Lobule IV/V female vs. Lobule X male | <0.0001 |
|  |  |  |  |  | Lobule IV/V female vs. Lobule X female | 0.0007 |
|  |  |  |  |  | Lobule IV/V female vs. Flocculus male | <0.0001 |
|  |  |  |  |  | Lobule IV/V female vs. Flocculus female | 0.0211 |
| Extended Data 1-3 b | Kruskal-Wallis test | H (5) = 12.43 | 0.0293 | 0.075 |  |  |
| Extended Data 1-3 c | Kruskal-Wallis test | H (5) = 79.49 | <0.0001 | 0.752 | Dunn’s multiple comparisons test |  |
|  |  |  |  |  | Lobule IV/V male vs. Lobule X male | <0.0001 |
|  |  |  |  |  | Lobule IV/V male vs. Lobule X female | <0.0001 |
|  |  |  |  |  | Lobule IV/V male vs. Flocculus male | 0.0004 |
|  |  |  |  |  | Lobule IV/V female vs. Lobule X male | <0.0001 |
|  |  |  |  |  | Lobule IV/V female vs. Lobule X female | <0.0001 |
|  |  |  |  |  | Lobule IV/V female vs. Flocculus male | 0.0007 |
| Extended Data 1-3 d | Kruskal-Wallis test | H (5) = 68.10 | <0.0001 | 0.751 | Dunn’s multiple comparisons test |  |
|  |  |  |  |  | Lobule IV/V male vs. Lobule X male | <0.0001 |
|  |  |  |  |  | Lobule IV/V male vs. Lobule X female | <0.0001 |
|  |  |  |  |  | Lobule IV/V male vs. Flocculus male | <0.0001 |
|  |  |  |  |  | Lobule IV/V female vs. Lobule X male | <0.0001 |
|  |  |  |  |  | Lobule IV/V female vs. Lobule X female | <0.0001 |
|  |  |  |  |  | Lobule IV/V female vs. Flocculus male | 0.0007 |
| Extended Data 6-1 a | Kruskal-Wallis test | H (2) = 3.546 | 0.1751 |  |  |  |
| Extended Data 6-1 b | Kruskal-Wallis test | H (2) = 10.95 | 0.0003 | 0.688 | Dunn’s multiple comparisons test |  |
|  |  |  |  |  | Lobule IV/V vs. Lobule X | 0.0414 |
|  |  |  |  |  | Lobule IV/V vs. Flocculus | 0.0061 |
| Extended Data 6-1 c | Kruskal-Wallis test | H (2) = 12.53 | <0.0001 | 0.810 | Dunn’s multiple comparisons test |  |
|  |  |  |  |  | Lobule IV/V vs. Lobule X | 0.0014 |
| Extended Data 6-1 d | Kruskal-Wallis test | H (2) = 12.92 | <0.0001 | 0.840 | Dunn’s multiple comparisons test |  |
|  |  |  |  |  | Lobule IV/V vs. Lobule X | 0.0011 |

Formulas used to calculate effect size:

**Ordinary one-way ANOVA:**

$$\omega^{2}=\frac{SSTreatment-\left( dfTreatment \times MSError \right)}{SSTotal+MSError}$$

SS: Sum of Squares

MS: Mean Square

**Kruskal-Wallis test:**

$$\eta_{H}^{2}=\frac{H-k+1}{n-k}$$

*H*: Kruskal-Wallis H-test statistic

*K:* Number of groups

*N:* Total number of observations

**Wilcoxon matched-pairs signed rank test:**

$$r=\frac{Z}{\sqrt{n}}$$

*Z*: Wilcoxon matched-pairs signed rank test Z statistic (Z statistic values obtained from SPSS)

*N:* Total number of observations

**Paired t test:**

$$Cohen’s d_{z} = \frac{t}{\sqrt{n}}$$

*t: t* value of the paired t test

*n:* Total number of observations
